# Use of a rapid digital microfluidics-powered immunoassay for assessing measles and rubella infection and immunity in outbreak settings in the Democratic Republic of the Congo

**DOI:** 10.1371/journal.pone.0278749

**Published:** 2022-12-21

**Authors:** Alaine K. Knipes, Aimee Summers, Alexandros A. Sklavounos, Julian Lamanna, Richard P. S. de Campos, Tanya Narahari, Christopher Dixon, Ryan Fobel, Yassa D. Ndjakani, Leopold Lubula, Alain Magazani, Jean Jacques Muyembe, Yvonne Lay, Elizabeth Pukuta, Diane Waku-Kouomou, Lijuan Hao, Jolie Kasongo Kayembe, Christian Fobel, Joshua Dahmer, Adam Lee, Man Ho, Jose Gilberto Camacho Valenzuela, Darius G. Rackus, Roger Shih, Brendon Seale, Ann Chang, Gilson Paluku, Paul A. Rota, Aaron R. Wheeler, Heather M. Scobie

**Affiliations:** 1 Division of Global Health Protection, Center for Global Health, Centers for Disease Control and Prevention, Atlanta, Georgia, United States of America; 2 Department of Chemistry, University of Toronto, Toronto, Ontario, Canada; 3 Donnelly Centre for Cellular and Biomolecular Research, University of Toronto, Toronto, Ontario, Canada; 4 Nanotechnology Research Centre, National Research Council of Canada, Edmonton, Alberta, Canada; 5 Sci-Bots Inc., Kitchener, Ontario, Canada; 6 Field Epidemiology and Laboratory Training Program, Center for Global Health, Centers for Disease Control and Prevention, Kinshasa, The Democratic Republic of the Congo; 7 Direction Générale de Lutte contre la Maladie, Ministry of Health, The Democratic Republic of the Congo, Kinshasa, The Democratic Republic of the Congo; 8 Institute National de Recherche Biomedicale, Ministry of Health, The Democratic Republic of the Congo, Kinshasa, The Democratic Republic of the Congo; 9 Division of Viral Diseases, National Center for Immunization and Respiratory Diseases, Centers for Disease Control and Prevention, Atlanta, Georgia, United States of America; 10 Programme Elargi de Vaccination, Ministry of Health, Kinshasa, The Democratic Republic of the Congo; 11 Institute of Biomedical Engineering, University of Toronto, Toronto, Ontario, Canada; 12 Routine Immunization and New Vaccine Introduction, World Health Organization, Libreville, Gabon; 13 Global Immunization Division, Center for Global Health, Centers for Disease Control and Prevention, Atlanta, Georgia, United States of America; CEA, FRANCE

## Abstract

The Democratic Republic of the Congo (DRC) has a high measles incidence despite elimination efforts and has yet to introduce rubella vaccine. We evaluated the performance of a prototype rapid digital microfluidics powered (DMF) enzyme-linked immunoassay (ELISA) assessing measles and rubella infection, by testing for immunoglobulin M (IgM), and immunity from natural infection or vaccine, by testing immunoglobulin G (IgG), in outbreak settings. Field evaluations were conducted during September 2017, in Kinshasa province, DRC. Blood specimens were collected during an outbreak investigation of suspected measles cases and tested for measles and rubella IgM and IgG using the DMF-ELISA in the field. Simultaneously, a household serosurvey for measles and rubella IgG was conducted in a recently confirmed measles outbreak area. DMF-ELISA results were compared with reference ELISA results tested at DRC’s National Public Health Laboratory and the US Centers for Disease Control and Prevention. Of 157 suspected measles cases, rubella IgM was detected in 54% while measles IgM was detected in 13%. Measles IgG-positive cases were higher among vaccinated persons (87%) than unvaccinated persons (72%). In the recent measles outbreak area, measles IgG seroprevalence was 93% overall, while rubella seroprevalence was lower for children (77%) than women (98%). Compared with reference ELISA, DMF-ELISA sensitivity and specificity were 82% and 78% for measles IgG; 88% and 89% for measles IgM; 85% and 85% for rubella IgG; and 81% and 83% for rubella IgM, respectively. Rubella infection was detected in more than half of persons meeting the suspected measles case definition during a presumed measles outbreak, suggesting substantial unrecognized rubella incidence, and highlighting the need for rubella vaccine introduction into the national schedule. The performance of the DMF-ELISA suggested that this technology can be used to develop rapid diagnostic tests for measles and rubella.

## Introduction

Measles and rubella (MR) cause a substantial public health burden and have been targeted for regional elimination. Measles caused an estimated 9.8 million cases globally in 2019, a 65% decrease from 2000 [1]. During 2000–2019, estimated measles deaths decreased 62% to 207,500, with an estimated 25.5 million deaths averted through vaccination [1]. Rubella typically causes mild illness, but infection during pregnancy can cause congenital rubella syndrome (CRS) leading to infant mortality and lifelong disability in surviving infants. Globally reported rubella cases decreased 96% to 26,006 during 2000–2018 [2]. Despite efforts and progress towards elimination, a global measles resurgence occurred in 2019, largely attributed to lack of vaccination [1]. In 2020, global emergence of COVID-19 further jeopardized routine immunization programs due to lockdowns and constrained health systems [3].

The Global MR Elimination Strategic Plan 2012–2020 outlined recommended strategies including high coverage with two doses of MR-containing vaccines, effective disease surveillance, outbreak preparedness and rapid response, and research and development of improved diagnostic tools [4]. Challenges with accurate assessment of subnational vaccination coverage to identify gaps in population immunity underscore the importance of effective measles surveillance to detect virus circulation and target interventions [5]. Measles and rubella are clinically similar fever-rash illnesses requiring laboratory testing for confirmation. The World Health Organization (WHO) recommends investigation of all suspected MR cases, often through integrated surveillance [5]; in the WHO African Region, only suspected cases testing negative for measles immunoglobulin M (IgM) are tested for rubella IgM. Challenges with consistent supplies for specimen collection and laboratory testing, specimen transport logistics, and laboratory capacity frequently occur in low-resource settings, resulting in suboptimal laboratory confirmation and uncertainty around disease burden and potential inappropriate targeting of limited program resources.

The Democratic Republic of the Congo (DRC) is a key country for measles elimination efforts in Africa because of its large population and geographic area, and central location with nine international borders [6]. In DRC, WHO and United Nations Children’s Fund estimates of first dose coverage of measles containing vaccine (MCV) provided at 9 months of age were steady at 57% during 2016–2019 [7]; a second MCV dose is only provided through vaccination campaigns, which often are delayed or attain suboptimal coverage (≤95%) [6]. National measles seroprevalence among children aged 6–59 months was assessed as 64% in DRC during 2013–2014 [8]. Notably, rubella containing vaccine (RCV) is not currently included in DRC’s Expanded Program for Immunization. Despite efforts towards measles elimination, large resurgences of measles have occurred in DRC during 2010–2013 [9] and 2015–2019 [1].

Laboratory support for MR surveillance is provided by the WHO Global MR Laboratory Network [10] which supports national laboratories in 191 countries including DRC. However, in many countries, improved MR diagnostic capabilities would be required to rapidly assess infection and immunity at the local level. A prototype of a new digital microfluidic (DMF) powered system for performing MR ELISAs in remote locations, the MR Box, was field tested in Kenya in 2016 [11] and included testing for immunoglobulin G (IgG) antibodies to assess population immunity from natural infection or vaccine and the related outbreak risk. A second-generation prototype of the DMF-ELISA (MR Box 2) was developed for a 2017 field test in DRC and included addition of IgM testing and portable battery capability, advancing its potential role as a rapid diagnostic test (RDT) in remote areas with limited access to centralized laboratories. The objectives of this study were to assess diagnostic performance of the DMF-ELISA for MR IgM and IgG in outbreak settings, including: 1) rapid confirmation of measles or rubella infection (IgM) in a suspected outbreak setting as part of active surveillance; 2) rapid assessment of MR population immunity (IgG) in a confirmed measles outbreak setting; and 3) comparing performance of DMF-ELISA to ELISA reference tests using outbreak specimens.

## Materials and methods

### Surveillance investigation

For the evaluation of DMF-powered immunoassay as a surveillance tool in a suspected outbreak setting, a team comprised of members from DRC’s Institut National de Recherche Biomedical (INRB), U.S. Centers for Disease Control and Prevention (CDC), and University of Toronto (U-Toronto), supported measles surveillance field investigations in Kinshasa province 30 August–23 September 2017. Members reviewed weekly district-level reports from Integrated Disease Surveillance and Response with the Directorate General for Disease Control and direct notifications through case-based surveillance. The team visited all facilities in Kinshasa health districts reporting suspected measles cases, defined as fever and maculopapular (non-vesicular) rash and either cough, coryza, or conjunctivitis, or an illness a health-care worker suspected to be measles [5]. Facility registers were reviewed to identify patients meeting the suspected case definition within two weeks prior to the visit by the team. Assuming 95% sensitivity and specificity of DMF-ELISA with a desired precision of +/- 7.5%, a final sample of 23 true MR IgM-positive specimens was required to compare performance of DMF-ELISA with reference ELISA. Assuming 25% of specimens would be IgM-positive, a sample size of 150 suspected cases was required to have a 90% probability of finding 23 or more true positive cases.

### Serosurvey

We conducted a household serosurvey 30 August–18 September 2017 in Biyela health zone, Kinshasa province, where a measles outbreak was confirmed in July 2017. The study area included an area surrounding the household of a confirmed measles case from the outbreak. Prior to the survey, a census of the study area was conducted 28–29 August 2017, in which all households with at least one eligible study participant (child aged 5–14 years or woman aged 15–49 years) were mapped and enumerated using tablets with EpiSample (PATH, MACEPA Developer Products, Seattle, WA). A household was defined as individuals who slept under the same roof the night before the visit. Compounds with multiple structures and heads of household were treated as separate households. Sample size was based on an estimated 60% rubella IgG seroprevalence with desired precision of +/- 8% separately for children aged 5–14 years and women aged 15–49 years. To have a 90% probability of obtaining desired precision, final sample sizes for each group were 100 children and 100 women, or 200 total individuals.

Using EpiSample, a random sample of 120 households (estimated number required to identify 100 children and 100 women) was selected from enumerated households in the study area; an additional 30 households were selected as backup if the sample size was not achieved from initial selection. Field team members used EpiSample to navigate to selected households. Three attempts were made to visit the 120 selected households before teams began visiting backup households. Any household with an eligible person was attempted for enrollment; where present within the same household, one woman and one child were randomly selected from among eligible women and children for enrollment.

### Data collection

Field teams, comprised of a medical epidemiologist with training in phlebotomy, an epidemiologist, and a local community health agent, visited households. For each household, a questionnaire was administered to each participant after obtaining informed consent (or from the responsible adult for a child) including name, address, phone number, age, sex, pregnancy status, any symptoms at time of data collection or in the previous 28 days, prior measles diagnosis, vaccination history, and contact history. Vaccination history was verified by vaccination card, if available. GPS coordinates were recorded for each household. A blood specimen was collected by venous puncture from each consenting individual and transported to the field lab for processing. All questionnaires, enrollment forms and blood specimens were labeled with unique barcodes to ensure lab results matched correctly with each participants’ data. This study received ethical approval from the DRC’s University of Kinshasa School of Public Health Ethical Committee. The Office of the Associate Director for Science at the Center for Global Health, U.S. Centers for Disease Control and Prevention (CDC) determined the evaluation not to be human subjects’ research and approved it as a public health program evaluation activity, according to U.S. Department of Health and Human Services Human Subjects regulations and procedures. Because the evaluation was not human subjects’ research, a consent procedure was approved where verbal consent was obtained from participants at the beginning of the survey as part of a standard script, and refusal was documented on forms by survey teams.

### Surveillance and serosurvey studies testing

Surveillance specimens were tested for MR IgG and IgM antibodies, while serosurvey specimens were tested for MR IgG only. U-Toronto team members tested all surveillance and serosurvey specimens on the same day as collection if possible, or within 48 hours, by DMF-ELISA in the field lab which was erected outdoors each day in the affected health district. The field lab consisted of DMF instruments (MR Box 2) and cartridges, portable tables and chairs, and a canopy to cover the work area from sun. A sample of whole blood was aliquoted for testing by DMF-ELISA, while the remainder of the blood specimen was separated into serum using a portable centrifuge. Sera were stored and transported to a centralized lab for testing using Siemens Enzygnost MR IgM ELISA kits (Siemens, Marburg, Germany) at INRB in Kinshasa, DRC and Zeus MR IgG ELISA kits (Zeus Scientific, Branchburgh, NJ, USA) at the CDC in Atlanta, USA; results were interpreted based on manufacturer specifications. Specimens sent to CDC for IgM testing were assessed using a Diamedix kit (Miami Lakes, FL, USA) for rubella IgM and a previously described CDC in-house measles IgM capture test [12].

### DMF devices, methods, and instrumentation

Magnetic-bead based immunoassays for four analytes (measles IgG and IgM and rubella IgG and IgM) and the MR Box 2 control system were designed and optimized in the laboratory in Toronto. Four MR Box 2 instruments and 1,000 cartridges were manufactured at U-Toronto and carried to DRC for the field trial. Positive and negative controls were included in each assay run and runs not meeting defined quality control criteria were excluded. DMF-ELISA results were normalized to account for differences in instrument, temperature, and humidity. Additional details are included in the S1 File.

### Analysis

Survey responses were collected on paper forms and entered in Epi Info version 7 (CDC, Atlanta, GA). Double data entry was performed, and data discrepancies reconciled by reviewing the paper forms. All analyses were conducted using STATA v15, Microsoft Excel 2013, Prism 8, and Python. Descriptive analyses were conducted, including proportions, means, and standard deviations, using reference ELISA results from INRB and CDC. Vaccine effectiveness (VE) during active surveillance was calculated as (1—[Odds cases vaccinated/ odds cases unvaccinated]). Performance of DMF-ELISA was assessed in comparison to reference ELISA, including receiver operating characteristic (ROC) analysis and calculation of sensitivity, specificity, and overall agreement of MR IgG and IgM results. Specimens collected from both surveillance and serosurvey were included in this comparison.

## Results

### Surveillance investigation

A total of 157 suspected measles cases were included in the surveillance study: among these, 86 (55%) occurred in female participants, and 90 (57%) in those aged <5 years. Measles IgM was detected in 20 (13%) cases; among these, 17 (85%) were <5 years of age, and three (15%) were 5–14 years. The proportion of suspected cases with detected measles IgM was highest among children aged <5 years (19%), compared with children aged 5–14 years (5%) and people aged ≥15 years (0%). Among 157 suspected cases, rubella IgM was detected in 84 (54%) cases; of which, 44 (52%) were among persons aged <5 years, 36 (43%) were 5–14 years, and four (5%) were ≥15 years. Proportions of suspected cases with detected rubella IgM were slightly higher in the age groups for ≥15 years (66%) and 5–14 years (59%), followed by <5 years (49%) (Table 1).

**Table 1:** Demographics, vaccination status, and measles and rubella IgM and IgG results among persons with suspected measles during an outbreak investigation, Kinshasa province, DRC, 2017.

	Total No. (Column %)	Measles IgM Positive No. (Row %)	Rubella IgM Positive No. (Row %)	Measles IgG^a^ Positive No. (Row %)	Rubella IgG^a^ Positive No. (Row %)
**Total**	157 (100)	20 (13)	84 (54)	134 (85)	110 (70)
**Sex**					
**Male**	71 (45)	9 (13)	34 (48)	61 (86)	45 (63)
**Female**	86 (55)	11 (13)	50 (58)	73 (85)	65 (76)
**Age**					
**<5 years**	90 (57)	17 (19)	44 (49)	72 (80)	52 (58)
**5–14 years**	61 (39)	3 (5)	36 (59)	56 (92)	52 (85)
**≥15 years**	6 (4)	0 (0)	4 (66)	6 (100)	6 (100)
**Measles vaccination status**					
**Vaccinated by card or recall**	118 (75)	8 (7)	64 (54)	103 (87)	88 (75)
**Not vaccinated**	29 (18)	12 (41)	10 (34)	21 (72)	12 (41)
**Don’t know**	10 (6)	0 (0)	10 (100)	10 (100)	10 (100)

^a^ There was insufficient sample quantity for the IgG testing of four measles specimens and 6 rubella specimens.

No. = Number. IgM = Immunoglobulin M. IgG = Immunoglobulin G.

Twenty-two (16%) persons reported a rash onset 0–3 days prior to sample collection; of these, three (14%) tested positive for measles IgM, and 11 (50%) tested positive for rubella IgM. One hundred six (76%) persons reported a rash onset 4–28 days prior to sample collection; of these, 12 (11%) tested positive for measles IgM and 59 (56%) tested positive for rubella IgM. Mean number of days reported since rash onset was 23 (**±** 2.3). Compared with confirmed rubella, a higher proportion of persons with measles IgM reported having fever (92% and 100%, respectively), runny nose (86% and 100%), cough (73% and 100%), or red eyes (67% and 90%) currently or within the previous 28 days; compared with confirmed measles, a higher proportion of persons with rubella IgM reported a history of swollen glands (35% and 81%, respectively).

Among 157 suspected cases, 134 (85%) were seropositive for measles IgG, and 110 (70%) were seropositive for rubella IgG. All 20 persons among whom measles IgM was detected were also seropositive for measles IgG, and 82 (98%) persons among whom measles IgM was detected were also seropositive for rubella IgG. Children with MR IgG detected were higher among ages 5–14 years (92% measles IgG and 85% rubella IgG) than <5 years (80% measles IgG and 58% rubella IgG); 100% of suspected cases among persons aged ≥15 years had detectable MR IgG, but the number of suspected cases in this group was small (n = 6). Persons with detectable rubella IgG were higher among females (76%) than males (63%), while persons with detectable measles IgG were similar by sex (85% and 86%).

Of 157 suspected measles cases, 118 (75%) reported having received MCV; however, only 15 (13%) had documented vaccination status. Among 118 persons with reported MCV, measles IgM was detected in eight (7%), while measles IgM was detected in 12 of 29 (41%) reported unvaccinated persons (Table 1). Twelve of 20 (60%) cases with detected measles IgM occurred in unvaccinated persons. Measles VE was estimated as 90% (95% CI, 71%–96%). Measles IgG-positive cases were higher among persons reporting vaccination (87%) versus unvaccinated (72%). Of the 15 persons with MCV documentation, none had detectable measles IgM, and 11 (73%) were measles IgG-positive; of the four without IgG-positive results, two were infants aged 10 months vaccinated a week prior to specimen collection, one was aged 3 years, and one had insufficient specimen volume for testing.

### Serosurvey

**Fig 1:**
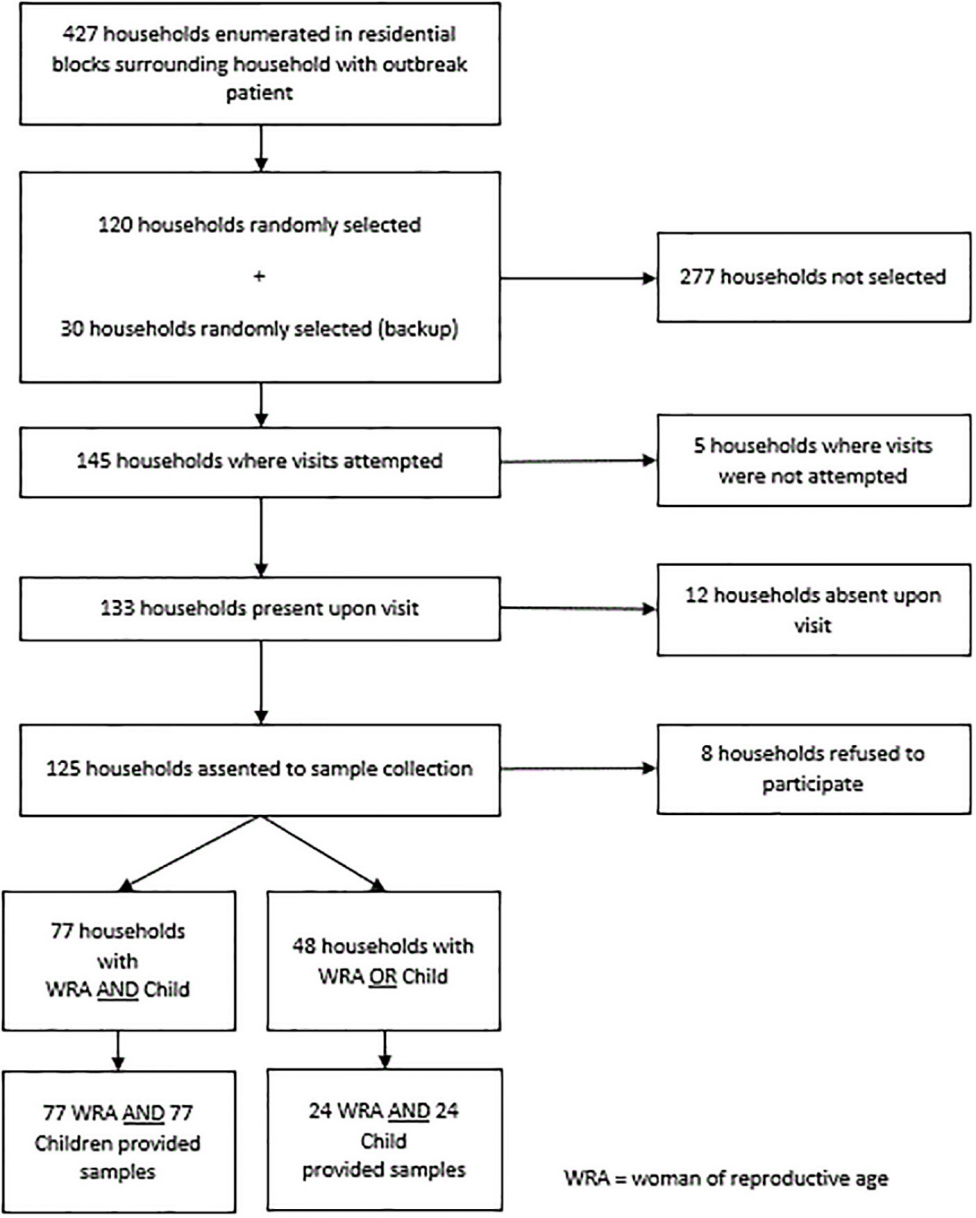
Serosurvey flowchart illustrating the number of households enumerated and study participants providing specimens.

A total of 427 households were enumerated in the survey area with a recent confirmed measles outbreak, and 145 households were visited for consent and enrollment. Of these, 125 (86%) consented to participate, eight (6%) refused, and 12 (8%) were absent (Fig 1). A total of 101 children and 101 women were enrolled in the seroprevalence survey. Mean ages of serosurvey participants were 9.5 (± 2.7) years for children and 28.0 (±8.5) years for women; among children, 57 (56%) were female (Table 2). Eighty-four (83%) children and 63 (62%) women reported receiving MCV, but only two children had vaccination cards available for verification. In both groups, measles IgG seroprevalence was 93%; no differences were observed in measles seroprevalence by vaccination status. Rubella IgG seroprevalence was 77% for children and 98% for women. Five (5%) of 101 women reported being pregnant; all five (100%) tested positive for rubella IgG, and four (80%) tested positive for measles IgG.

**Table 2:** Demographics, measles vaccination status, and measles and rubella IgG results for serosurvey—Biyela health district (one month after confirmed measles outbreak), Kinshasa province, DRC, 2017.

	Children (5–14 years) [n = 101)	Women (15–49 years) [n = 101]
**Mean age in years (standard deviation)**	9.5 (2.7)	28.0 (8.5)
	**No. (%)**	**No. (%)**
**Sex**		
**Male**	44 (44)	NA
**Female**	57 (56)	101 (100)
**Measles vaccination status**		
**Vaccinated (by card or recall)**^a^	84 (83)	63 (62)
**Not vaccinated against measles**	4 (4)	5 (5)
**Don’t know**	13 (13)	33 (33)
**Measles IgG ELISA**		
**Positive**	94 (93)	94 (93)
**Negative**	4 (4)	5 (5)
**Equivocal**	3 (3)	2 (2)
**Rubella IgG ELISA** ^b^		
**Positive**	78 (77)	99 (98)
**Negative**	22 (22)	2 (2)
**Equivocal**	1 (1)	0 (0)

^a^ Only two children aged 5–14 years had cards available to verify vaccination status. No women 15–49 years had vaccination cards available to verify their history of vaccination from childhood.

^b^ One sample had insufficient quantity for rubella testing.

No. = Number. NA = Not applicable. IgG = Immunoglobulin G. ELISA = enzyme-linked immunoassay.

### Performance characteristics of DMF-ELISA

The MR Box 2 instrument, method, and cartridges featured a long list of advances relative to what was previously described [11], including the capacity to test four analytes (measles IgG and IgM and rubella IgG and IgM), and the ability to operate from battery power (S1 File).

**Fig 2:**
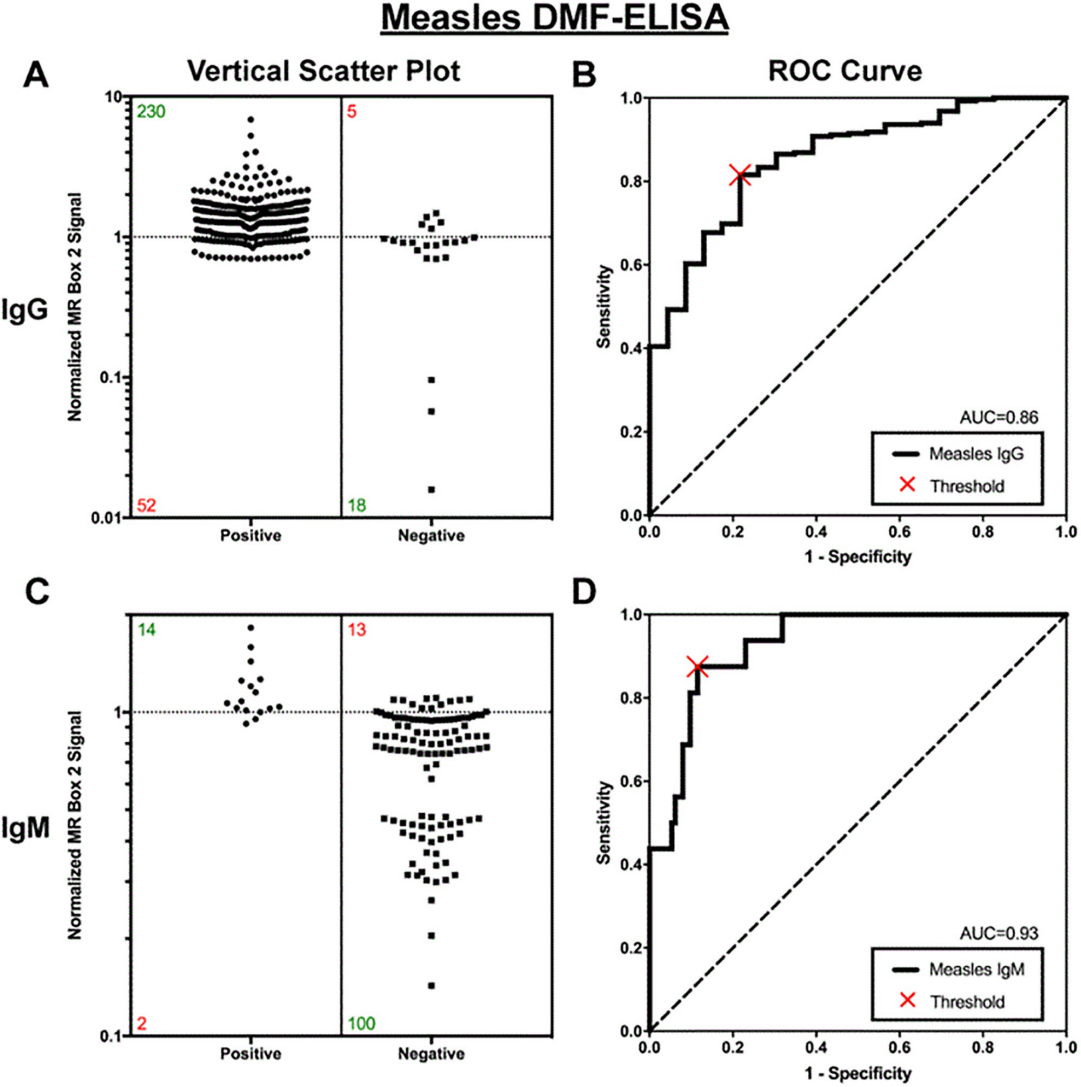
Performance of DMF-ELISA for assessing 305 specimens for measles IgG (A and B) and 129 specimens for measles IgM (C and D) compared with a reference ELISA. (A and C) Vertical scatterplot (left) of MR Box 2 signals for specimens determined to be positive or negative for anti-measles IgG (top, n = 305) and IgM (bottom, n = 129) by reference tests. Green and red numbers in the scatterplot represent the number of specimens correctly and incorrectly categorized by the MR Box 2, respectively. (B and D) Receiver Operating Characteristic (ROC) curves with an optimized threshold (X).

In the comparison of specimens tested on-site using the MR Box 2 with reference testing at INRB and CDC, a total of 14.5% of test specimens (148 out of 1,017 specimens used for testing) were excluded for not meeting quality control standards. For measles IgG specimens (n = 305), the area under the curve (AUC) for ROC analysis was 0.86. Compared with reference ELISA, sensitivity of the measles IgG DMF-ELISA was 82% (95% CI, 77%–86%), specificity was 78% (95% CI, 56%–93%), and overall agreement was 81% (95% CI, 76% –86%). For measles IgM specimens (n = 129), the AUC was 0.93, and sensitivity, specificity, and agreement were 88% (95% CI, 62%–98%), 89% (95% CI, 81%–%–94%), and 88% (95% CI, 82%–93%), respectively (Fig 2).

**Fig 3:**
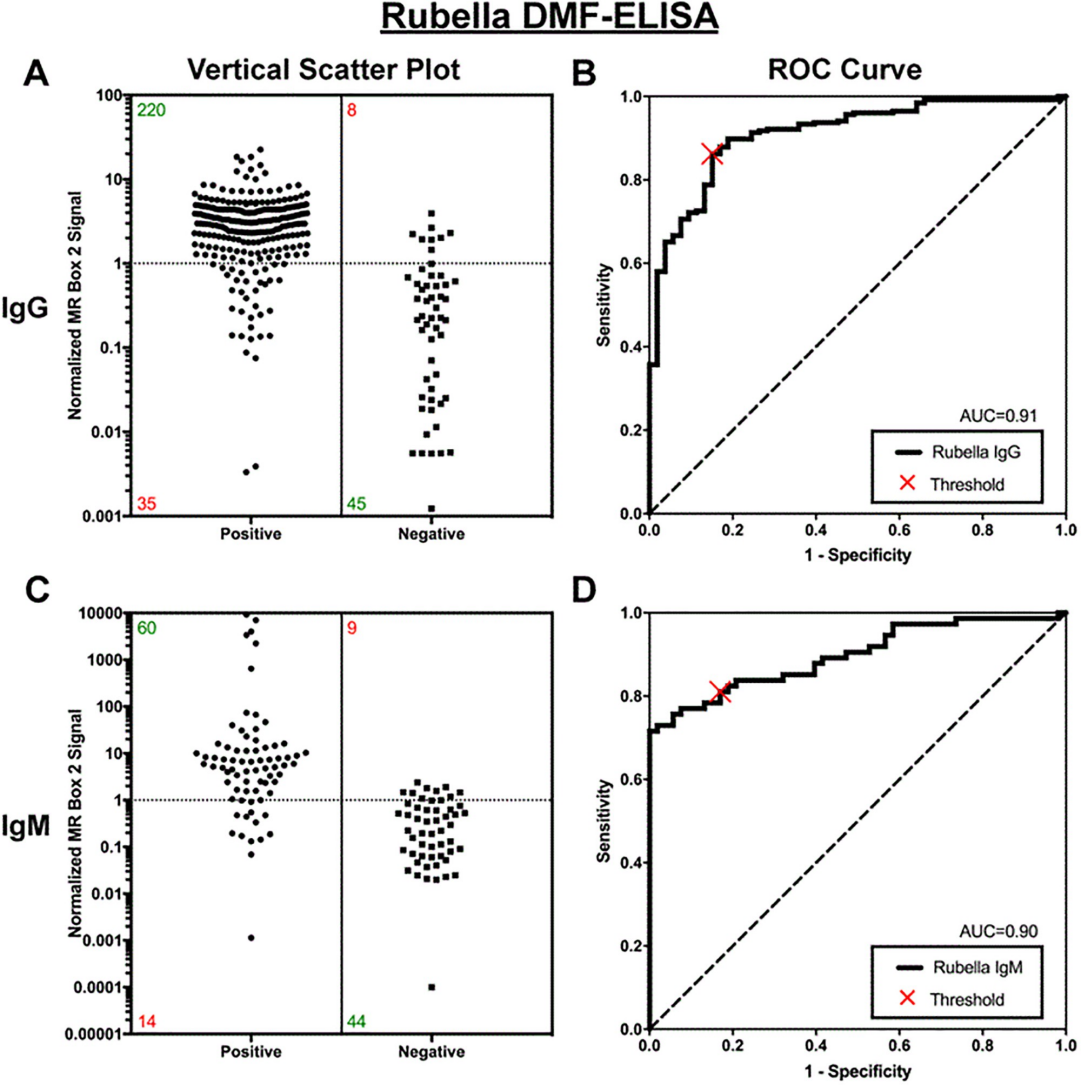
Performance of DMF-ELISA for assessing 308 specimens for rubella IgG (A and B) and 127 specimens for rubella IgM (C and D) compared with a reference ELISA. Vertical scatterplot (left) of MR Box 2 signals for specimens determined to be positive or negative for anti-rubella IgG (top, n = 308) and IgM (bottom, n = 127) by reference tests. Green and red numbers in the scatterplot represent the number of specimens correctly and incorrectly categorized by the MR Box 2, respectively. (B and D) Receiver Operating Characteristic (ROC) curves with an optimized threshold (X).

For rubella IgG specimens (n = 308), the AUC was 0.91, sensitivity was 86% (95% CI, 81%–90%), specificity was 85% (95% CI, 72%–93%), and overall agreement was 86% (95% CI, 82%–90%) (Fig 3). For rubella IgM (n = 127) specimens, the AUC was 0.90, and sensitivity, specificity and agreement were 81% (95% CI, 70%–89%), 83% (95% CI, 70%–92%), and 82% (95% CI, 74%–88%), respectively (Fig 3).

## Discussion

This study in DRC found that upon serologic confirmation of suspected measles cases, most infections were caused by rubella, similar to previous reports [13]. Without strong case surveillance including lab-confirmation, monitoring progress towards measles elimination is made more challenging by circulating rubella, with potential to inappropriately direct MCV vaccine and other limited public health resources in response to rubella outbreaks. In DRC, evidence of the relative burden of circulating rubella and epidemiologic evidence of affected age groups from our field investigation in Kinshasa support the need for RCV introduction.

Confirmed measles cases with detectable measles IgM were more likely to be young (85% aged <5 years) and unvaccinated against measles (60%), indicating gaps in DRC’s vaccination program. Previous assessments in DRC identified suboptimal measles protection and observed deficiencies in routine immunization, including failure to vaccinate and reduced VE [8, 14–16]. VE in our outbreak investigation in Kinshasa was high (90%, 95% CI, 71%–96%), supporting under-vaccination as the likely outbreak cause. VE was higher than a previous national estimate of 80% (95% CI, 74%–85%) among ages 12–59 months in DRC, though a higher risk of measles was noted in provinces other than Kinshasa, which could be related to documented challenges with vaccine logistics and cold chain in areas outside the capital [14].

Seroprevalence for measles was 93% for both children and women, which was likely a combination of both protection from vaccination and natural infection related to the recently confirmed measles outbreak in the area. While only 83% of children and 62% of women reported vaccination against measles, it is difficult to precisely determine the contribution of immunization to high measles seroprevalence, as recall of vaccination status is generally unreliable [17], especially for older children and adults. Antibody avidity testing to determine recent or past IgG response was not attempted.

Among those in whom rubella IgM and IgG were detected during active surveillance, a majority (60% and 59%, respectively) were female, and nearly half (48%) of those with detected rubella IgM were aged >5 years. A difference in rubella IgG seroprevalence was observed between children (77%; mean age = 10 years) and adult women (98%; mean age = 28 years) during the serosurvey. With a lack of rubella vaccination and a high proportion of persons with rubella IgM detected among older ages groups, young women reaching adulthood in DRC may be left at higher risk of infection during pregnancy and higher CRS risk for their infants [18]. This further highlights the need for RCV introduction through routine childhood immunization and a wide age-range campaign targeting persons up to age 15 years to catch-up those not protected through natural infection [19].

The first use of the prototype DMF-ELISA for MR IgG testing in Kakuma Kenya during 2016 demonstrated a potential role for the diagnostic tool in remote areas with limited access to centralized laboratories [11]. Following additional development and lab testing at U-Toronto, the latest DMF-ELISA prototype was deployed for field validation in Kinshasa, DRC including additional MR IgM testing capabilities. The 81%–88% agreement for DMF-ELISA for IgG and IgM testing in the DRC field validation was promising for a prototype and similar to the percent agreement in the Kenya field validation of the DMF-ELISA for IgG testing (84%-86%), but highlighted the need for additional optimization before considering the test for routine field use. For example, DMF cartridges bearing pre-deposited, dried reagents [20] might be useful to improve reproducibility in future studies. Field validation was useful in identifying environmental conditions (temperature and humidity) that needed to be adjusted for in the results. An existing lateral flow-based RDT for measles IgM detection has been field-validated with high sensitivity and specificity compared to ELISA, and this test is being commercialized, along with commercial development of a rubella IgM RDT; these tests have the potential use by non-expert users as part of MR surveillance [21]. Compared to a lateral flow device, the advantages of the DMF-ELISA include the ability to readily print cartridges at low cost and the ability to customize the system *ad-hoc* for use with different assay configurations based on need.

This study had some limitations. First, the serosurvey excluded children aged <5 years due to challenges with collecting blood intravenously from small children; this would have been a valuable age group to assess for measles seroprevalence. Second, DMF-ELISA testing was performed by expert users, and results were not available in real time due to the need for subsequent adjustment and interpretation of results; the ELISA results used in the investigation and presented here were not impacted by this limitation. Third, participant recall was relied upon for history of vaccination and clinical symptoms, but accurate recall likely varied with time from the event.

A high percentage of people with suspected measles in in this outbreak setting were found to have rubella (54%), highlighting the unrecognized burden of rubella in this community and the need for better diagnostics to investigate outbreaks of rash illness. Additionally, lower rubella IgG seroprevalence occurred in older children, and a high proportion of cases with detectable rubella IgM occurred in older age groups, raising concerns around CRS risk for young women. The Kinshasa outbreak investigation documented high VE against measles among suspected measles cases, confirming that gaps in vaccination were the likely outbreak cause. The serosurvey documented high measles seroprevalence in a community that had recently experienced a measles outbreak. Findings from our field investigation in Kinshasa supported the need for introduction of RCV in DRC and further assessments in other countries that have not introduced RCV and have frequent outbreaks of suspected measles.

## Supporting information

S1 File (PDF)
