# Supplementary material for: Use of a rapid digital microfluidics-powered immunoassay for assessing measles and rubella infection and immunity in outbreak settings in the Democratic Republic of the Congo
Source: PLoS One. 2022 Dec 21;17(12):e0278749. doi: 10.1371/journal.pone.0278749 (PMC9770344; doi:10.1371/journal.pone.0278749)
Supplement: S1 File — (PDF) [file pone.0278749.s001.pdf]

Supporting Information

Contents

Supplementary Methods 2
- Materials and Reagents 2
- Clinical Samples 3
- DMF Cartridge Fabrication 3
- DMF Cartridge Quality Control 5
- MR Box v2 5
  - DMF Electronics 5
  - Peripheral Electronics 6
  - Peripheral Hardware 6
  - Enclosure 7
  - Peripheral Software 7
- Benchmark for Bead Retention 7
- DMF Assay Development and Optimization 8
  - Conjugate Stability Optimization 9
- DMF Assay Validation 10
  - Effect of Temperature on the Chemiluminescence Signal 10
- Field Trial Data Acquisition and Analysis 11
  - Normalization and Analysis 12
- MR Box v2 and Cartridges 13
- Assay Development, Optimization, and Validation 15
- Field Trial 18

Supplementary Figures 21

Supplementary Tables 32

Supplementary References 37

Supplementary Methods

Materials and Reagents

Unless otherwise specified, reagents were purchased from Sigma-Aldrich, and electronic components were purchased from Digi-Key. Deionized water with a resistivity of >18 megaohm•cm was used to prepare all aqueous solutions. Three types of solutions, including (i) Rubella Virus (RV) IgG and IgM standards, (ii) RV IgM pretreatment reagent containing goat anti-human IgG, and (iii) RV virus coated paramagnetic particles (5.0-µm-diameter) were adapted from the Architect™ RV IgG and IgM assay kits (Abbott Laboratories). Measles Virus (MV) IgG and IgM standards were prepared from anti-measles serum (WHO Third International Standard, UK National Institute for Biological Standards and Control). Reagents from other vendors included SuperBlock™ Tris-buffered saline, Guardian™ stabilizing solution, SuperSignal™ ELISA Femto chemiluminescent substrate [comprising stable hydrogen peroxide (H_2O_2) and luminol/enhancer solution], and the Pierce™ TMB Substrate Kit [comprising 3,3',5,5' tetramethylbenzidine (TMB) and hydrogen peroxide (H_2O_2)] from Thermo Fischer Scientific, and ethylenediamine tetrakis(ethoxylate-block-propoxylate) tetrol (Tetronic 90R4) from BASF Corp.

The reagents for the DMF-ELISAs (reported here for the optimized protocol, see relevant section below) were formulated as follows: (i) wash buffer (WB) was Dulbecco's phosphate-buffered saline (DPBS) supplemented with 0.1% w/w Tetronic 90R4, (ii) blocking diluent was SuperBlock™ supplemented with 0.1% w/w Tetronic 90R4, (iii) sample (and assay control) diluent was DPBS supplemented with 4% (w/w) bovine serum albumin (BSA) and 0.1% w/w Tetronic 90R4, and (iv) conjugate diluent was Guardian™ solution. IgM pretreatment working solution was formed from a 3× dilution of the stock RV IgM pretreatment reagent in sample diluent. IgG and IgM conjugate working solutions were anti-human IgG–peroxidase (A8792) produced in rabbit (100 ng/mL) and anti-human IgM– peroxidase (A6907) produced in goat (100 ng/mL) in conjugate diluent. Luminol/enhancer and stable peroxide aliquots were each supplemented with 0.05% (w/w) Tetronic 90R4. Positive control solutions for the four assays were

(i) calibrant F from the Architect™ RV IgG kit (75 IU/mL), (ii) calibrant 1 from the Architect™ RV IgM

kit (50 IU/mL), (iii) WHO MV IgG standard (50 IU/mL), and (iv) WHO MV IgM standard (50 IU/mL), all diluted in sample diluent. Negative controls for (ii-iv) were the same solutions diluted to <10 IU/mL; the negative control for (i) was calibrant A from the Architect™ RV IgG kit (0 IU/mL). Measles virus coated paramagnetic particles were prepared using 3.07-μm-diameter amine-terminated paramagnetic particles (Bangs Laboratories) which were sensitized with inactivated Edmonston strain measles virus (catalog #7604, Meridian Life Science Inc.) using a method described elsewhere.[1] Before use, particles for both MV and RV assays were washed twice with blocking diluent and resuspended in blocking diluent at 9×10^8 (MV) or 1.5×10^8 (RV) particles/mL.

Clinical Samples

Two types of samples were used for method development in Toronto prior to the field trial. (1) Blood was collected from healthy volunteers in Toronto by venipuncture in Vacutainer® K_2EDTA tubes (BD) (10 mL) or by finger-prick in Microtainer® K_2EDTA tubes (BD) 0.5 mL following Protocol #: 00034019, which was reviewed and approved by the University of Toronto Research Ethics Board (REB). (2) Plasma samples from subjects in the DRC were shipped to Toronto and were received and processed following Protocol #: 00034991, which was reviewed and approved by the University of Toronto REB. Samples evaluated in the DRC field trial are described in the main text.

DMF Cartridge Fabrication

DMF cartridges comprised two plates (bottom and top). Bottom plates (76.2 mm × 76.2 mm × 1.1 mm) bearing 110 individually addressable electrodes were fabricated using either photolithography or inkjet printing. Bottom plates fabricated photolithographically were generated from Cr-coated glass slides (Telic Company, CA) using methods described previously.[2] Bottom plates fabricated by inkjet printing were formed using an Epson C88+ inkjet printer (Seiko Epson Corporation) to pattern Metalon JS-B25P silver nanoparticle ink onto Novele IJ-220 substrates (NovaCentrix), as described previously.[3] After printing, bottom plates were diced and affixed to glass slides (S.I. Howard Glass Co. Inc.) with double-sided adhesive tape (3M, MN). After patterning (by photolithography or printing), all bottom plates were coated with

Parylene C and FluoroPel PFC 1104V (Cytonix LLC), as described previously.[4] As a final step, a strip of copper tape (3M, MN, part no. 9876-15) was adhered to the ground pad to facilitate electrical connection to the top-plate upon assembly.

The DMF bottom plate design was constructed in AutoCAD (Autodesk, CA) and featured an array of 78 offset-cross- shaped actuation electrodes (2.8 mm × 2.8 mm), six rectangular mixing electrodes (5.7 mm × 2.4 mm), six square waste electrodes (2.8 mm × 2.0 mm), 10 rectangular dispensing electrodes (5.2 mm × 2.4 mm), and 10 reservoir electrodes (10 mm × 6.7 mm). The gap between the electrodes was set at 100 µm to allow the design to be printed using a low-cost inkjet printer. Each electrode was connected via a patterned wire that interfaced with an array of electrode pads on the side of the bottom plate. A unique cartridge batch ID was imprinted on the bottom plate at the fabrication to enable batch tracking and cartridge quality control.

Most ("standard") DMF top plates were formed from indium tin oxide (ITO)-glass slides (25 mm × 75 mm × 0.7 mm, 8-12 Ω/sq, Riley Supplies, ON, Canada). Some ("PET-ITO") DMF top plates were formed by adhering glass microscope slides (25 mm × 75 mm × 1.0, Fisherbrand) to pieces of indium tin oxide coated polyethylene terephthalate (PET) film (60 Ω/sq) cut to match the slides, via clear double-sided adhesive tape (Adhesives Research Inc., PA, part no. ARcare 93551). The ITO sides of both types of top plates were coated with FluoroPel PFC 1104V as described previously,[3] Each cartridge was assembled by joining the top and bottom plate with two layers of Scotch double-sided tape (3M), which served as a spacer creating an inter-plate gap of approximately 200 µm. After assembly, a treatment with 3M Scotchweld 2-part epoxy (3M) was used to permanently affix the two plates together. Unit droplets were approximately 1.1 µL, and reservoir electrodes could be loaded with approximately 7.5 µL. More than 1500 cartridges were fabricated and used for method optimization and testing including 1000 cartridges dedicated to the field trial. The latter included 500 photolithographically defined bottom plates assembled with standard top plates, 100 photolithographically defined bottom plates assembled with PET-ITO top plates, and 400 inkjet printed bottom plates assembled with standard top plates.

DMF Cartridge Quality Control

Each batch of assembled cartridges (~30/batch) was subjected to a quality control assessment to assess the performance of the batch, indicate cartridge flaws, and determine the appropriate operation voltage. The assessment comprised two steps. First, a pair of unit-droplets of wash buffer were driven across all the electrodes of the cartridge to ensure that no pinholes where present in the dielectric and no hydrophilic spots were found on either the bottom or top plate. Second, a capacitance test was performed to determine the appropriate voltage for the batch of cartridges required to generate a driving force of 25 mN/m, a value that was found empirically to be below the saturation velocity.[5] This step was crucial to ensure cartridge longevity and successful completion of the DMF assays.

MR Box v2

MR Box v2 was designed and manufactured with custom components and functions for the current project, comprising DMF electronics, peripheral electronics, peripheral hardware, an enclosure, and peripheral software (summarized in S1 Table and described in detail below). All custom printed circuit boards (PCBs) in the system were designed using the open-source software tool KiCad (https://gitlab.com/kicad) and manufactured by PCBway (China).

DMF Electronics

The DMF controller was built with OEM components from the open-source DropBot v3 platform (Sci-Bots, ON, Canada). The system consisted of a main control board (handling high-voltage AC signal generation, impedance sensing, and communication with the host PC), three high-voltage switching boards supporting up to 120 independent opto-isolated channels, and a pogo-pin connector board that provided the electrical interface with the DMF chip. Power (+12V and +3.3V) and digital communication (I^2C) signals were distributed to all electronics submodules in the instrument via a custom front panel board. The DMF electronics were periodically tested using a hardware testing board, comprising a series of 120 10 pF capacitors, aligned such that each connected to one of the 120 actuation channels of the control system.

Peripheral Electronics

A custom peripheral control board that interfaced with the DropBot electronics (above) was built to control the peripheral devices in MR Box 2, including the photomultiplier tube (PMT), the pump, the Z-stage, and the temperature/ humidity sensor. The board included an ATMEGA328 (Microchip Technology Inc.) microcontroller, an A4988 stepper motor driver (Pololu, NV) to control the Z-stage stepper, and a Honeywell HIH6000 temperature and humidity sensor. The board communicated directly with the host computer using a USB to TTL serial adapter (USB BUB II, Modern Device, RI).

Another custom "PMT board" was built to quantify the current produced by the PMT and to transmit the readings to the peripheral board. The PMT board included a transimpedance amplifier (LMP7721, Texas Instruments), a digital potentiometer (MCP41010, Microchip Technology) and an Analog-to-Digital Converter (MAX11210, Maxim Integrated). The board included through-holes matching the pins of the PMT which allowed direct connection of the board and the PMT in the MR Box v2. The PMT board was powered by the peripheral board and exchanged data using a ribbon cable (Digikey, MN). A "PMT test board" was also developed which mimicked a DMF device, comprising contact pads, RGB light emitting diodes (LEDs) (SMLP34RGB2W3, Digikey) and MOSFETs (SSM3K339R, Digikey). The board was used as a standard DMF cartridge, with the only difference being that MR Box 2 actuated the channels with 3V DC and the light emitted was used to test PMT function and to calibrate and optimize the location of the detection region on the DMF cartridge.

Peripheral Hardware

A PMT with gated function (H12056-110, Hamamatsu) was attached to a milled Delrin spacer and was affixed to the MR Box 2 chassis facing the top side of the DMF cartridge. Two aspherical condenser lenses (ACL1210U-A, Thorlabs Inc.) were mounted in a 3D-printed PLA housing printed with an Ultimaker 2 (Ultimaker, Netherlands), positioned between the PMT and the cartridge. During operation the PMT was biased at 1000 V.

A magnetic lens with shape and dimensions described previously [1] was affixed to a custom, stepper-motor

controlled Z-stage (assembled from parts in S2 Table) that was integrated into the MR Box v2 to move the lens to and away from the DMF cartridge for magnetic bead pulldown. The Z-stage had a vertical travel range of 25 cm and comprised 3D printed PLA parts, metal parts and a stepper.

A USB8MP02G-L75-CA (ELP, Amazon.ca) web camera was mounted on the lid of MR Box 2 using a 3D printed PLA holder. A panel of 24 White LEDs (White LED 24 SMD 3528 Panel, Amazon.ca) was affixed to the side of the lid adjacent to white cardstock to diffuse and reflect light.

Finally, a 11000mAh 12V battery pack (YB12011000-USB, TalentCell) was attached at the back of the MR Box v2 and was used to power the instrument. Each MR Box v2 measured 25.4 cm × 19 cm × 23 cm (w × l × h)., weighed ~4.5 kg, and a total of four boxes were built and used at the field trial in the DRC.

Enclosure

The instrument's ruggedized case was built from two off-the-shelf NEMA enclosures customized with machined cut- outs, mounting holes, and silkscreen labels (Polycase, OH). The base was made from a diecast aluminum enclosure (AN-23P, Polycase) and housed the magnetic separation stage, DMF electronics and peripherals. A plastic enclosure (YH-080604) was bolted to the top of the base allowing for a light-tight, hinged lid that provided easy access to the DMF chip. This lid also housed the webcam and PMT.

Peripheral Software

A custom plugin for the DMF control software Microdrop (version 2.19, https://github.com/sci-bots/microdrop) was developed in Python that enabled control of all the peripheral devices from the main user interface.

Benchmark for Bead Retention

Suspensions of RV-modified (5-µm-diameter), MV-modified (3-µm-diameter) and Dynabeads MyOne Streptavidin T1 (1-µm-diameter, Thermo Fisher) paramagnetic particles were prepared in wash buffer in various densities. Beads were loaded into a DMF cartridge operated using the MR Box or the new MR Box 2 (using the new magnetic Z-stage) and distributed between four bead trapping regions on the cartridge. The

magnet was actuated, and the wash buffer was removed leaving behind a pellet of particles on the trapping region. The capacity for each box to capture the beads was recorded by observing the formed pellet. In cases of "failed retention" the pellet was pulled by the moving droplet away from the trapping region. Measurements were performed in triplicates.

DMF Assay Development and Optimization

Samples for development and optimization experiments were formed from whole blood collected from volunteers in Toronto. Immediately after collection, the plasma was replaced by centrifuging (5 min, 1,500 × g), removing the supernatant, and gently resuspending the red blood cell pellet in plasma analogue (PBS + 4% BSA) at a volume equal to that of the discarded supernatant. This process was repeated twice more (for a total of three replacements) to yield antibody depleted whole (ADW) blood. Positive and negative samples were formed by spiking ADW blood with standards to have concentrations equivalent to the positive and negative control solutions (described above) and were stored at 4°C until use. In most cases, the optimized DMF ELISA protocol was used (defined below); in other cases, a design of experiments (DOE) optimization was carried out, varying some of the variables, as summarized in S3 Table.

The optimized DMF ELISA is as follows. A sample or control was manually diluted 1:5 for RV IgG and IgM, and MV IgM assays, and 1:10 for MV IgG assays in sample diluent. For IgM assays, the diluted samples or controls were then manually mixed with pretreatment solution (anti-human IgG, 2:1 sample to pretreatment) and incubated for seven minutes. Sample or control and reagents were then loaded onto a cartridge, which was inserted into MR Box v2 and each assay was performed in 17 automated steps. (1) A double-unit droplet containing paramagnetic particles (coated with rubella inactivated virus for anti-rubella assays or measles inactivated virus for anti-measles assays) was dispensed from a reservoir and separated from the diluent. (2) A double-unit droplet of sample was dispensed and mixed with the particles for seven minutes. (3) The particles were immobilized, and the supernatant droplet was driven to waste. (4-9) The particles were washed six times, in each case resuspending in a fresh double-unit droplet of wash buffer, immobilizing the particles, and driving the supernatant droplet to waste. (10) A double-unit droplet of

conjugate solution (anti-human IgG conjugate or anti-human IgM conjugate pre-diluted to 100 ng/mL) was dispensed and mixed with the particles for five minutes. (11-14) The particles were washed four times as above. (15) A unit droplet of luminol was dispensed, used to resuspend the particles, and moved to the detection region. (16) A unit droplet of H_2O_2 was dispensed and mixed with the droplet containing the suspended particles for one minute. (17) The chemiluminescence signal was recorded by the PMT (biased at 1000 V) for 100 seconds (one sample/sec). The average PMT response during this period was recorded as "signal" and the suspension was driven to waste. Typically steps 1- 14 of the procedure above were applied in parallel to five solutions (often three samples, a positive and a negative control). Steps 15-17 were then performed serially for each of the solutions until completed. Note that a blank measurement was also made (identical to the "signal" in step 17, but with no droplet in the detection zone), at the beginning of each assay. This value was either subtracted from the signal after the assay or used to calibrate the PMT's ADC zero point prior to the assay (steps that were functionally equivalent).

Conjugate Stability Optimization

Stock solutions of IgG and IgM assay secondary antibodies (antibody-peroxidase conjugate) were separately diluted 1:100k (IgG) or 1:10k (IgM), in SuperBlock™, Guardian™ solution, or StabilZyme™ HRP Conjugate Stabilizer (Surmodics IVD Inc.). These solutions were aliquoted into individual tubes and stored in heat-sealed mylar silver packaging at different temperatures (4°C, 22°C, or 37°C). Aliquots were tested on days 0, 7, 14, and 30 and the signals were normalized to those generated from aliquots stored at 4°C. For testing, 100 µL aliquots of diluted RV IgG standard (Abbott Architect™ kit, 5 IU/mL in carbonate buffer pH 9.5) or diluted RV IgM cut-off standard (Abbott Architect™ kit, 1:10 dilution in carbonate buffer pH 9.5) were added to each well of a 96-well plate and incubated overnight at 4°C for passive functionalization. Next, the standard was discarded, and each well was washed 3X with 200 µL of PBST (PBS with 0.05% w/w Tween 20). The plates were blocked with 200 µL of SuperBlock™ for one hour at room temperature, and washed 3X with 200 µL of PBST. 50 µL of each IgG or IgM conjugate (freshly prepared or after storage) was added to each functionalized, blocked well and incubated for one hour at

room temperature on a shaker. After incubation, the wells in the plate were washed 3X with 200 µL of PBST. A 100 µL solution of 1:1 mixture of TMB and peroxide was added to each well and incubated for 15 minutes at room temperature. The reaction was stopped by adding 100 µL of sulfuric acid (1 M). Absorbance measurements were obtained at 450 nm and reference at 650 nm using a Sunrise microplate reader (Tecan).

DMF Assay Validation

Samples used for validation experiments were either (i) whole blood samples collected in Toronto (collected and stored at 4°C until use), or (ii) plasma samples from DRC subjects shipped to Toronto in dry ice and then stored at - 80°C until use. The former samples were evaluated using the optimized DMF ELISA protocol immediately after collection ("raw"), and also after converting them into ADW blood and spiking them to be negative for IgG (as described above). The latter samples were evaluated using the optimized DMF ELISA protocol without modification. For comparison, these samples were also evaluated (separately) by commercial reference test for MV IgM, as described in the main text. Upon unblinding, MR Box v2 results were aligned with reference results and a blank- subtracted PMT-response threshold was selected to differentiate between samples found to be positive or negative by the reference tests.

Effect of Temperature on the Chemiluminescence Signal

IgG assay secondary antibodies (antibody-peroxidase conjugate) were diluted to 1 ng/mL in Guardian™ solution. An MR Box 2 was placed in a lab oven (DX302C, Yamato Scientific America Inc.) and the temperature was set at 24° (oven off), 32°, 36° or 40°C and left for 30 minutes for the temperature to equilibrate. Then a new DMF cartridge was inserted in the box and loaded with the conjugate solution or a blank solution (Guardian ™ solution only) and a 1:1 mixture of luminol and H_2O_2 solutions. A unit droplet of each reagent was dispensed from their reservoirs, merged, and mixed for one minute, and then the chemiluminescence signal was recorded as in step (17) above. Replicate signals were averaged, blank-subtracted, and then normalized to the average blank-subtracted signal for the 24°C condition. Normalized values were plotted as a function of temperature and a centered second order polynomial (quadratic) equation was fitted to the data [$Y= 1.359 + 0.1050*X_C + 0.0072*X_C^2$ where $X_C = X – X_{mean}$ (X_{mean}

= 33°C).

Field Trial Data Acquisition and Analysis

Field trial tests were carried out from 30 August to 25 September 2017. Assay reagents were stored at 4°C overnight and aliquots of each were carried into the field (at ambient temp.) each day. Four MR Box 2 instruments were used to run assays using the optimized protocol described above, recording PMT signals for each sample plus a positive and a negative control, as well as metadata, including (a) Date, (b) Location, (c) Box ID, (d) Laptop, (e) Operator, (f) Cartridge Type, (g) Top-plate Type, (h) Cartridge ID, (i) Analyte, (j) Temperature, (k) Relative Humidity, (l) Number of Standards (Negative / Positive), and (m) Operator's Comments. In all, 157 whole blood samples were evaluated from the surveillance investigation (given sample IDs ranging between DRC-I-1 and DRC-I-200) and 202 from the household serosurvey (given sample IDs ranging between DRC-S-201 and DRC-S-450).

In initial tests in the field trial, solutions prepared in Toronto were used as positive and negative controls (as planned). But beginning 5 September 2017, a rotating series of replacement controls (comprising plasma from samples found in MR Box v2 experiments to have 'high' or 'low' signal from a recent/previous day and then stored at stored at 4°C) were used for some tests. Specifically, MV IgG assay positive controls used on 5-13 September, 13 September, 14-15 September, and 16-25 September were plasma from samples DRC-S-212, DRC-S-239, DRC-I-062, and DRC-S-257, respectively, and MV IgG negative controls used on 6-15 September and 16-25 September were plasma from samples DRC-S-211 and DRC-S-302, respectively. Likewise, MV IgM positive controls used on 6-16 September and 18-25 September were plasma from samples DRC-I-004 and DRC-I-045, respectively, and MV IgM negative controls used on 6-16 September, 18-21 September, and 21-25 September were plasma from samples DRC-I-010, DRC-I-071 and DRC-I-041, respectively. Finally, RV IgM positive controls used on 15-24 September were plasma from sample DRC- I-067.

A total of 1,017 such measurements (353 MV IgG, 156 MV IgM, 351 RV IgG and 157 RV IgM) were collected and measured during the course of the field trial. 110 measurements (44 MV IgG, 7 MV IgM, 43

RV IgG and 28 RV IgM) were excluded from the final analysis on the basis of operator's comments (e.g., observation of excessive electrolysis, hardware malfunction, etc.), eight measurements (four MV IgG, two MV IgM, and two RV IgM) were excluded because the sample PMT signal was below a pre-determined cut-off threshold (1.0×10^{-9} a.u.), and 18 measurements (MV IgM) were excluded because the positive control to negative control signal ratio was less than 1.6 (tested prior to adopting the rotating controls described above). The remaining 869 measurements (305 MV IgG, 129 MV IgM, 308 RV IgG and 127 RV IgM) were grouped according to some of the abovementioned metadata (Box ID, Cartridge Type, Temperature, Relative Humidity) and sample signals were normalized as described below.

Normalization and Analysis

A series of multiplicative and subtractive factors (fm and fs respectively, S4 Table) were found to relate Box ID, cartridge type, temperature, and relative humidity such that the sets of positive controls and negative controls in each type of assay were equivalent. Equation 1 was used to normalize MV IgG and IgM signals while Equation 2 was used to normalize RV IgG and IgM signals.

$$Normalized\ Signal = \left(Signal * f_{m,_{Box\ ID}} * f_{m,_{Humidity}}\right) - f_{s,_{Temperature}}$$

Equation 1. Normalization function for MV IgG and IgM signal.

$$Normalized\ Signal = \left(Signal * f_{m,_{Cartridge\ Type}}\right) - f_{s,_{Cartridge\ Type}}$$

Equation 2. Normalization function for RV IgG and IgM signal.

MR Box 2 signals normalized according to Equations 1 and 2 were aligned with gold standard results and processed using the following Python packages; SciPy[6], Numpy[7], Pandas[8], Scikit-learn[9]. Vertical Scatter and Receiver Operating Characteristic (ROC) plots were drawn using GraphPad Prism 7 (GraphPad Software Inc.). Confidence Intervals were calculated according to the binomial distribution.

MR Box v2 and Cartridges

In a 2018 publication[1], we introduced a portable digital microfluidic system known as the measles-rubella box (or MR Box) that was used to run ELISAs for measles virus (MV) and rubella virus (RV) IgG in pin-prick blood samples in the Kakuma refugee camp in Kenya in a field trial in 2016. The MR Box v1[1] system was useful, but had a number of limitations, including (most importantly) the requirement of external power. To support the field trial in the DRC reported here, we developed a radically redesigned system known as the "MR Box v2," shown in S1A Fig., featuring a custom peripheral board that manages all of the box functionalities and collection of assay data for analysis.

The most important new feature of MR Box v2 is the option to run on either an integrated/detachable 12 V battery pack or a 12V power supply. The former comfortably allowed for continuous operation for a day of work (with >12 hours battery lifetime) without the need for electrical mains, which was critical for the DRC field trial (described below), in which tests were run outdoors with no access to AC power. Beyond the battery pack, there were a number of additional upgrades that were required for the DRC field trial, including improved detector reproducibility, an improved device I/O interface, and an improved magnetic stage for bead pulldown (each addressed briefly below). Four identical MR Box v2 instruments were built and transported to DRC for the field trial. As indicated in S1 Table, the cost of goods (CoGs) to form each system was $5,616 USD (United States Dollars).

A key limitation of the MR Box v1[1] was a lack of measurement reproducibility between the different instruments, which was attributed to variations in PMT (i) dark current, (ii) signal noise, and (iii) position. To address (i), as illustrated in S1B Fig., a PMT with a gating function (an electronic shutter) was incorporated in MR Box v2, to allow the PMT to always remain powered on. This configuration permits

dark current to settle[10], in contrast to MR Box v1 in which the PMT was continually powered on and off as the box was opened and shut for device insertion and removal. To address (ii), the new PMT in MR Box v2 featured a custom board matching the pin footprint of the PMT, featuring a transimpedance amplifier, a digital potentiometer to set the PMT bias current, and an analog-to-digital converter to allow immediate conversion of the PMT signal to a digital signal (minimizing loss during signal transmission). Finally, to address (iii), the PMT and the lens-pair system were permanently mounted above the top of the DMF cartridge, which was found to have greater position-stability than the PMT in MR Box v1 that was mounted on the lid of the box (and thus was regularly moved). Finally, as illustrated in S2 Fig., a custom detector-calibration board (a PCB bearing an array of LEDs) was used to map the optimal electrode/droplet locations for light collection, to ensure uniform performance from each of the PMTs in the MR Box v2 systems.

The MR Box v1[1] (and most other DMF control systems) uses an array of pogo-pins to make high-voltage connections with DMF cartridges. Mating devices to pogo-pins can be 'tricky', requiring operator expertise and adjustment-time to address each of the tiny contact pads with the correct pin (e.g., in MR Box v2, there are 136 pads distributed into two arrays, each with dimensions 8.89 × 41.91 mm). This is acceptable for small numbers of experiments in the lab but was found to be a critical shortcoming for the Kenya field trial, in which hundreds of samples were evaluated in sub- optimal circumstances in a short period of time.[1] To overcome this challenge for MR Box v2 and the DRC field trial, we developed a new Push to Load (P2L) mechanism (S1D Fig.), for fast, reliable, and seamless loading and unloading of the devices on the box. Briefly, P2L relies on a plastic slider that mates with the device and inserts into the box, automatically aligning contact pads to pogo-pins. Using the P2L, a novice user can insert a device in ~2 seconds, making a secure and stable connection without failure.

Finally, MR Box v1[1] featured a rotating servo motor to actuate a magnetic lens into position for magnetic bead pulldown in sample droplets. This system was found to have two key limitations that were not acceptable for the project described here. (i) The rotation mechanism of the magnetic lens in MR Box v1[1] did not allow for fine control of the magnet’s position. As a result, the only tenable 'magnet engaged' position (that did not risk colliding with devices) was ~mm away from the sample droplets, which reduced

the strength of the magnetic field at the trapping region, limiting the range of pull-down operations that could be carried out. (ii) Servos such as those used in MR Box v1[1] require continuous supply of power (whether in “magnet engaged” or “magnet disengaged” position), which would result in depleting the battery if used in the MR Box v2. To overcome these limitations, a new vertical Z-stage was designed and used in the MR Box v2 (S1D Fig.). As shown, the Z-stage relies on a stepper motor which is engaged only when the magnet lens is moving (overcoming limitation ii above). Furthermore, the improved precision of the Z-stage allows reproducible positioning of the magnetic lens to be a few microns from devices (with no risk of collision (overcoming limitation I above). To quantify the effect of the latter improvement, bead capture experiments were tested head-to-head on the two instruments (MR Box v1[1] and 2). As shown in S3 Fig., the Z-stage outperforms the servo actuator in bead retention and repeatability of the capture. Importantly, not only was the performance of the Z-stage better than the alternative, but also the Z-stage was economical to build (<$35 USD CoGs per system, S2 Table).

In sum, the battery-pack-powered MR Box v2 represents a complete re-design-and-implementation relative to its predecessor – an advance that was necessary for the demanding requirements posed by the assays and field trial, described below. In contrast, the cartridges used in the current field trial were quite similar to those used previously.[1] The one exception was the test of a new form of "PET-ITO" top-plate, which reduced the cost from $6 USD/cartridge for the Kenya field trial [1] to less than $2.50 USD for the CoGs to form a printed bottom plate and a PET-ITO top plate in the experiments described here. A head-to-head comparison between the key differences of the two systems is also summarized in S5 Table.

Assay Development, Optimization, and Validation

As was the case for the previous field trial in Kenya,[1] in the work reported here, digital microfluidic immunoassays were developed, optimized, and validated in a laboratory in Toronto before implementing in the field. Like the previous study, the new assays were automated, paramagnetic particle-based ELISAs. But the new assays differed from the previous assays in two key respects. First, unlike the previous study (which included only IgG tests to report long-term immune status), the new study featured both IgG and

IgM tests (incl. four different assays, testing for MV and RV IgG and IgM), to report both long-term immune status and recent infection status, as illustrated in S4A Fig. Second, the new tests were developed and optimized using a matrix that was a closer match to the samples (whole blood) tested in the field. Specifically, the samples used to develop and optimize were formed from blood collected from volunteers in Toronto. But because these samples nearly uniformly have high IgG titer (because of vaccination), in optimization studies, the red blood cells (RBCs) were isolated and washed, and then resuspended in immunoglobulin-free plasma substitute. These antibody-depleted whole (ADW) blood samples were then spiked with known amounts of an IgG or IgM standard, which was used for all optimization steps. The use of spiked ADW blood samples was an advance, as we speculated previously[1] that the assays used in Kenya (which were developed using only plasma samples) may have been poorly optimized for the whole-blood matrix tested in the field.

Upon development of the essential assay reagents and materials for the four assays, a design of experiments (DOE) model was implemented to identify the final procedures used in lab- and fieldwork. Four variables were tested: the level of sample dilution (off-chip, prior to loading onto the cartridge), the amount of time in which the sample was incubated with the paramagnetic beads (on-chip), the number of bead-wash steps implemented after sample incubation (on-chip), and the level of conjugate solution dilution (off-chip, prior to loading onto the cartridge). As indicated in S3 Table, a definitive screening design[11] (DSD) was applied, in which three variables were optimized for the RV IgG assay, and two variables each for the MV IgG, the RV IgM, and the MV IgM assays, respectively (reducing the total number of experiments populating the DOE from 54 to 18). As illustrated in S4B Fig., the DSD data were fit using a second order model, evaluating the significance of individual variables and the interactions between multiple variables. Sample data from these experiments are shown in S4C-E Fig., and from the results, the following conventions were adopted for the optimized protocol – a 1:5 sample dilution for RV IgG, RV IgM, and MV IgM assays and a 1:10 sample dilution for MV IgG assays, a seven-minute sample-particles incubation (all assays), six post-incubation wash steps (all assays), and dilutions of the stock conjugate solutions to 1:100,000 (for IgG assays) or 1:10,000 (for IgM assays). The final method comprised 17 automated steps and required ~70

minutes to complete to evaluate three samples and two controls from beginning to end.

Armed with an optimized assay protocol, we moved to developing a robust stabilization strategy for the assay reagents, recognizing that there would be no temperature control in the field trial. The most delicate reagent was thought to be the secondary antibody-enzyme conjugate – thus, with limited time available for this work, our efforts were focused on identifying conditions that maintain the activity of this reagent after exposure to elevated temperatures. Three stabilizing solutions were evaluated – SuperBlock™ (the conjugate diluent used in the Kenya field trial[1]), Guardian™ solution (a commercial peroxidase conjugate stabilizer/diluent advertised to preserve diluted conjugates for more than six months at room temperature without significant loss of activity) and StabilZyme™ solution (a commercial peroxidase conjugate stabilizer advertised to protect the entire conjugate by preventing the loss of catalytic activity and maintaining the structural integrity of the protein in solution). As shown in S5 Fig., both Guardian™ and StabilZyme™ were significantly better than SuperBlock™ at preserving peroxidase activity when stored at temperatures above 4°C. The same reagents were then evaluated for their effects on the optimized digital microfluidic RV IgG and IgM assays. As shown in S6 Fig., using Guardian™ or StabilZyme™ as conjugate stabilizers did not significantly affect the assay performance relative to SuperBlock™ in the IgG assay. In contrast, in the IgM assay, both Guardian™ and StabilZyme™ caused statistically significant reductions in chemiluminescent signal for the positive ADW blood sample measurements. These reductions (~18% for Guardian™ and ~38% StabilZyme™) were small and did not interfere with the ability to discriminate between positive and negative samples. Given the great improvement in long-term stability, it was decided that the signal decrease was tolerable. Finally, noting that fluid characteristics like viscosity and surface tension can have dramatic effects on droplet velocity in DMF,[5] the same reagents were tested for their effects on droplet movement. As shown in S7 Fig., droplets of Guardian™ had velocity profiles similar to wash buffer and SuperBlock™, while droplets of StabilZyme™ were substantially more sluggish. Guardian™ was thus selected as the conjugate diluent and was used in all experiments described below.

After completing the optimization experiments, a subset of the methods (in limited time prior to the field trial) were evaluated in two sets of validation experiments. First, a new set of whole blood samples (from

vaccinated volunteers) were collected and evaluated (blind) before and after the antibody depletion process. As shown in S8 Fig., the tests perfectly discriminated "high" from "low" IgG concentration in this small sample set. Second, a set of plasma samples obtained from the DRC was evaluated by the DMF MV IgM assay, compared to reference test results (S9 Fig). As shown, the DMF MV IgM assay had good agreement with the reference test results, yielding a receiver operator characteristic (ROC) curve with 92% sensitivity and specificity and an area under the curve (AUC) of 0.95. This performance was deemed satisfactory, giving the team confidence to embark on the field trial, described below.

Finally, noting that all of the optimization and validation experiments were carried out in the laboratory (with relatively constant temperature and humidity), and that diagnostic assays relying on chemiluminescence are notoriously sensitive to variations in these parameters,[12] preliminary experiments were run to assess the effect of temperature for the assays used here. As shown in S10 Fig. the chemiluminescent signal catalyzed by the conjugate used in the optimized assays is highly dependent on temperature, which suggested that a sophisticated normalization scheme might be needed for the field trial, in which temperature and humidity were not controlled.

Field Trial

As described in the main text, four MR Box v2 instruments and 1,000 DMF cartridges were used to carry out a field trial in DRC. In a typical day, the team (i) left the hotel, carrying the instruments, cartridges, and a day's worth of assay reagents that had been stored overnight at 4°C, (ii) set up a mobile testing site outdoors, (iii) collected blood samples from subjects, (iv) used a portable centrifuge to form plasma to freeze and ship for reference testing, (v) tested a small aliquot of whole blood on the MR Box v2 on-site (in parallel with iv), and (vi) returned to the hotel (which had AC power), allowing the battery packs to recharge overnight.

Two general challenges were encountered during the field trial. First, as indicated in S11 Fig., the temperature and humidity varied substantially from day to day as recorded by the MR Box v2 sensors (i.e., from <25°C to >45°C and from <30% to >80%, respectively). As indicated above (and as illustrated in S10 Fig.), temperature variations in this range are expected to have substantial effect on the signal

measured. Secondly and more perniciously, as time passed, the measurement reproducibility of some of the positive and negative control solutions were found to diminish with time (assumed to be an effect of reagent 'spoilage'). In an attempt to compensate for this unexpected problem, a rotating series of control-solution replacements was used for some assays (recorded in detail in the Supporting Methods section), comprising plasma from samples found on a recent day to have "high" or "low" signal in an MR Box v2 assay to serve as temporary positive and negative controls. These were then 'rotated' to new plasma samples as the original controls were used up. Note that the stabilized conjugate reagents did not appear to have the spoilage that was observed for the positive/negative controls; in hindsight it is clear that we should have developed a similar approach to stabilization of the controls (using a protein stabilizing solution) as was applied to the conjugate (S5 Fig.). In the future, an even more robust approach will be to use stabilized, dried reagents, which can be stored for months at a time with minimal sensitivity to temperature (as described recently for a different DMF application[13]).

In addition to the general challenges (which affected all assays), a ~15% assay failure-rate was observed for the field trial, in which the cartridge did not work properly, or the instrument malfunctioned in some way, as well as the first set of spoiled-control experiments, before the rotating control replacements were applied. But the vast majority of the MR Box v2 tests – some 869 measurements (including 305 MV IgG, 129 MV IgM, 308 RV IgG and 127 RV IgM) – were found to have run successfully (on whole blood samples in the field). These results were evaluated relative to the (separate and subsequent) reference test results (on frozen, then thawed plasma samples in the laboratory).

To compensate for the general challenges indicated above (variations in temperature and humidity and a rotating set of non-uniform control solutions), mathematical relationships were derived (Equation 1 and Equation 2) to normalize the signals generated from the positive and negative controls in each type of assay on the basis of differences in instruments, humidity, temperature, and cartridge type (S4 Table). This type of normalization has been used[14, 15] in laboratory experiments to evaluate large datasets involving measurements collected over long durations and has been proposed to be useful for portable assays[16]. Most importantly, this scheme was required to accommodate the (unexpected and non-ideal) series of

rotating control solutions that were used in the field. In sum, these corrections were applied to the 869 measurements, which were then compared to reference test results to generate the scatterplots and receiver operator characteristic (ROC) curves shown in Figs 4 and 5 in the main text. As indicated in the main text, the field-test results and the reference test results matched relatively well (with AUCs of 0.86-0.93 in the ROC curve analyses). Drilling down, no trend of dis/concordance was observed for the different instruments or device types; they all had similar performance relative to the reference tests.

Supplementary Figures

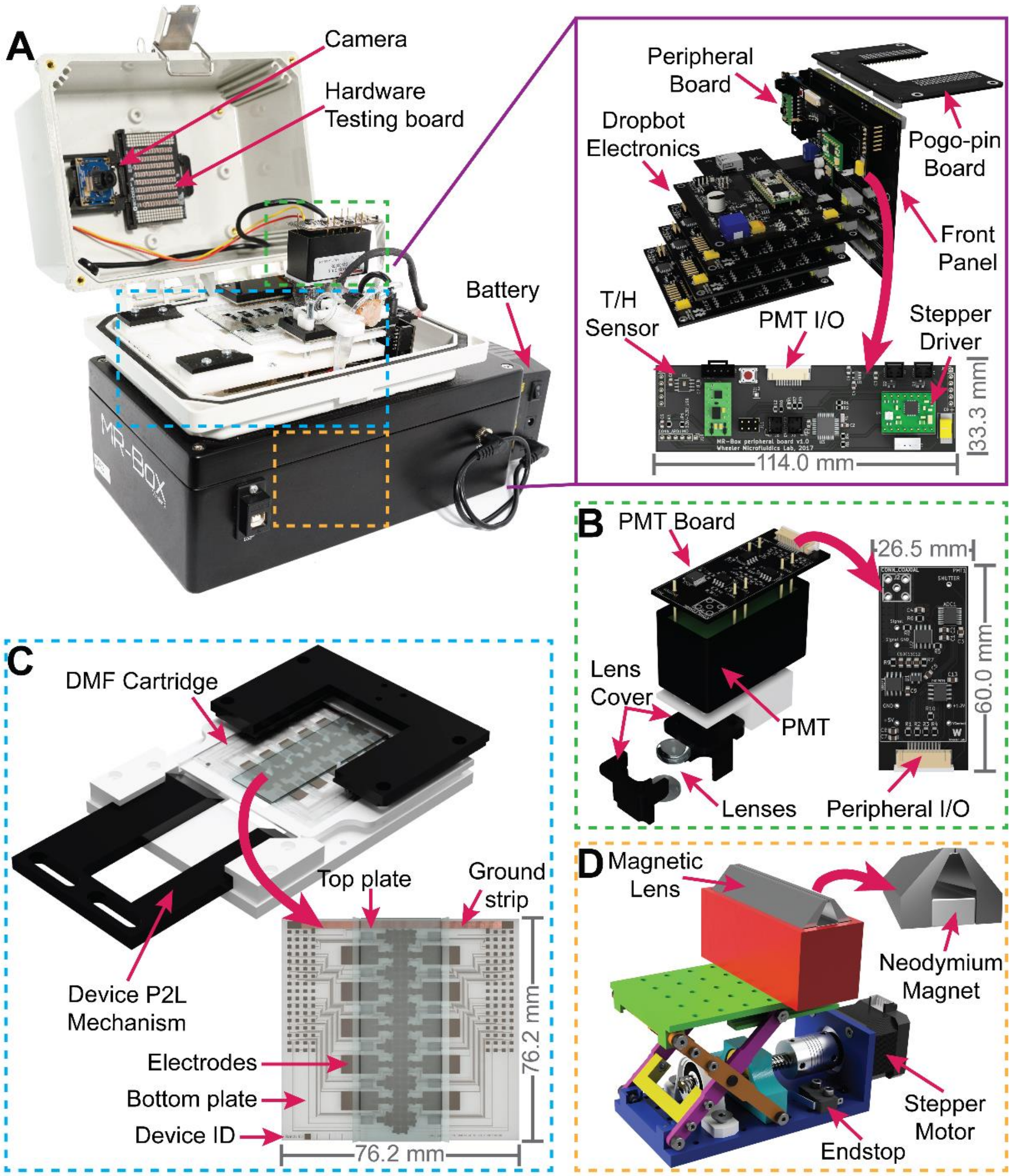

S1 Fig. MR Box v2 and digital microfluidic cartridge. (A) Main: photograph of the MR Box v2 (24 cm × 19 cm × 21.5 cm) showing the battery pack, webcam, and hardware testing board. Dashed boxes indicate the photomultiplier tube (PMT, green), chip loading dock (blue), and magnetic Z-stage (yellow). Call-out: digital rendering of the key MR Box v2 electronics, including the DropBot boards used to control droplet movement, the pogo-pin connector for powering the DMF cartridge, and a peripheral board that controls a stepper driver, a temperature and humidity (T/H) sensor, and the PMT. **(B)** Digital rendering of the PMT, the focusing lens pair, the lens housing and the custom board designed to power the PMT, control the shutter and measure the low currents produced when the PMT is exposed to light. The PMT board has a connection port that allows connection to the peripheral board. **(C)** Digital rendering of the new chip loading mechanism (Push to Load, P2L) and the DMF cartridge. **(D)** Digital rendering of the custom motorized z-stage used to actuate the magnetic lens.

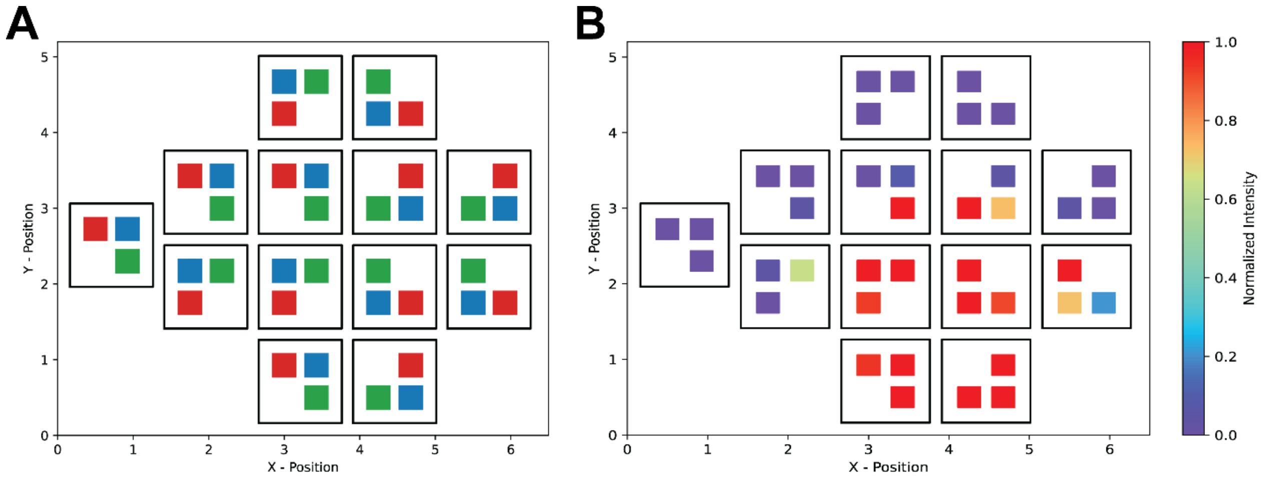

S2 Fig. MR Box v2 PMT-position calibration. (A) Position-map of tricolor LEDs (Red, Blue, Green) on the PMT calibration board, designed to emulate chemiluminescent droplets on DMF cartridges. (B) Example of a position-map of normalized light intensity (shown as a heat-map from low-purple to red-high) measured in an MR Box v2 instrument to calibrate PMT/cartridge positioning.

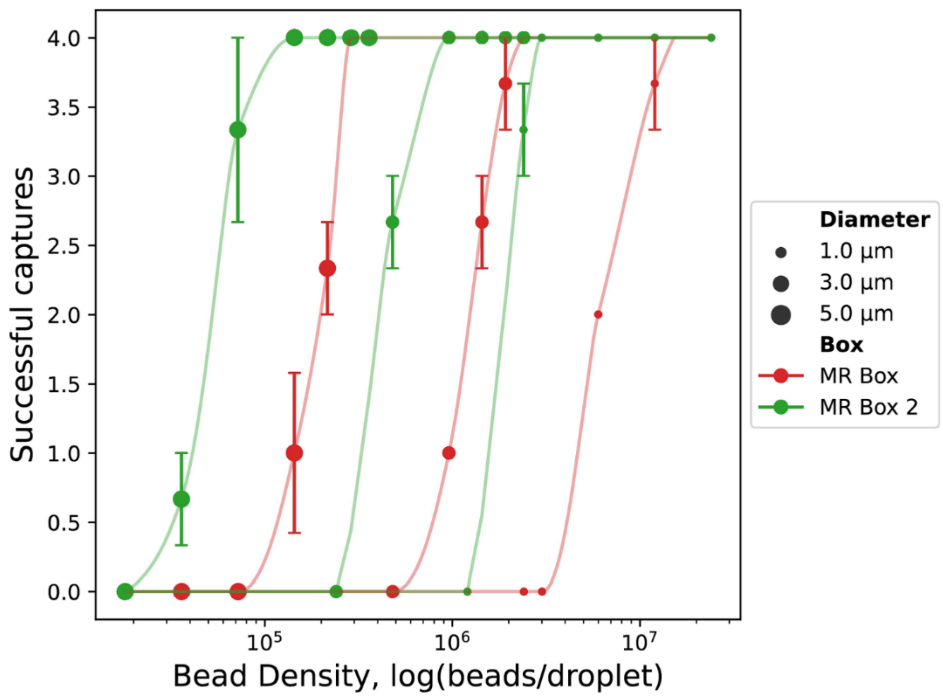

S3 Fig. Comparison between MR Box and MR Box 2 bead capturing capabilities. Plots of magnetic bead capture success observed as a function of the logarithm of bead suspension density for the MR Box v1[1] with servo/rotation stage (red) and MR Box v2 with stepper motor/Z-axis stage (green). In each experiment, beads were captured from four droplets in parallel and each outcome was rated for bead capture (1 = success; 0 = failure), such that value of 4.0 reports a "perfect" capture from all droplets. The size of spots represents the bead diameter (1.0, 3.0, 5.0 µm diameter). Error bars represent std. dev. from n = 3 measurements per conditions.

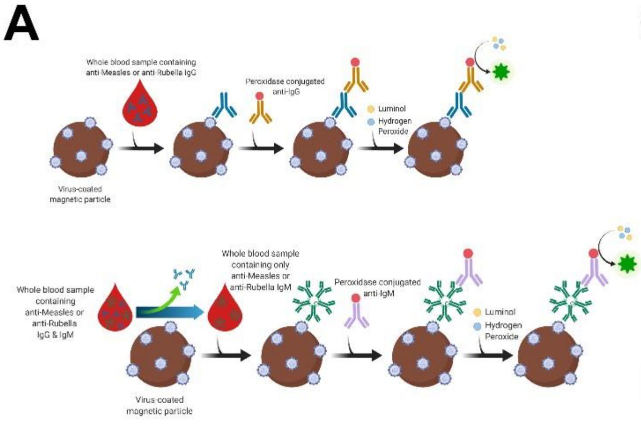

B

Term	Estimate	Std Error	t Ratio	Prob>|t|
Concentration[0]	-2.761e-6	1.524e-7	-18.11	<.0001*
Intercept	4.2216e-6	2.988e-7	14.13	<.0001*
Concentration[0]*Wash Steps	-1.112e-6	1.735e-7	-6.41	<.0001*
Incubation Time*Dilution	8.7537e-7	1.773e-7	4.94	<.0001*
Incubation Time(3,7)	6.8987e-7	1.418e-7	4.87	<.0001*
Dilution(0.05,0.2)	7.5565e-7	1.611e-7	4.69	0.0001*
Dilution*Dilution	-8.654e-7	2.653e-7	-3.26	0.0036*
Dilution*Concentration[0]	-6.221e-7	1.94e-7	-3.21	0.0041*
Wash Steps(2,6)	4.9018e-7	1.872e-7	2.62	0.0157*
Wash Steps*Wash Steps	5.0338e-7	2.323e-7	2.17	0.0414*
Incubation Time*Concentration[0]	-1.749e-7	1.392e-7	-1.26	0.2223
Incubation Time*Wash Steps	-2.166e-7	1.789e-7	-1.21	0.2388
Incubation Time*Incubation Time	1.6625e-7	2.356e-7	0.71	0.4878
Dilution*Wash Steps	-1.145e-7	2.683e-7	-0.43	**0.6737**

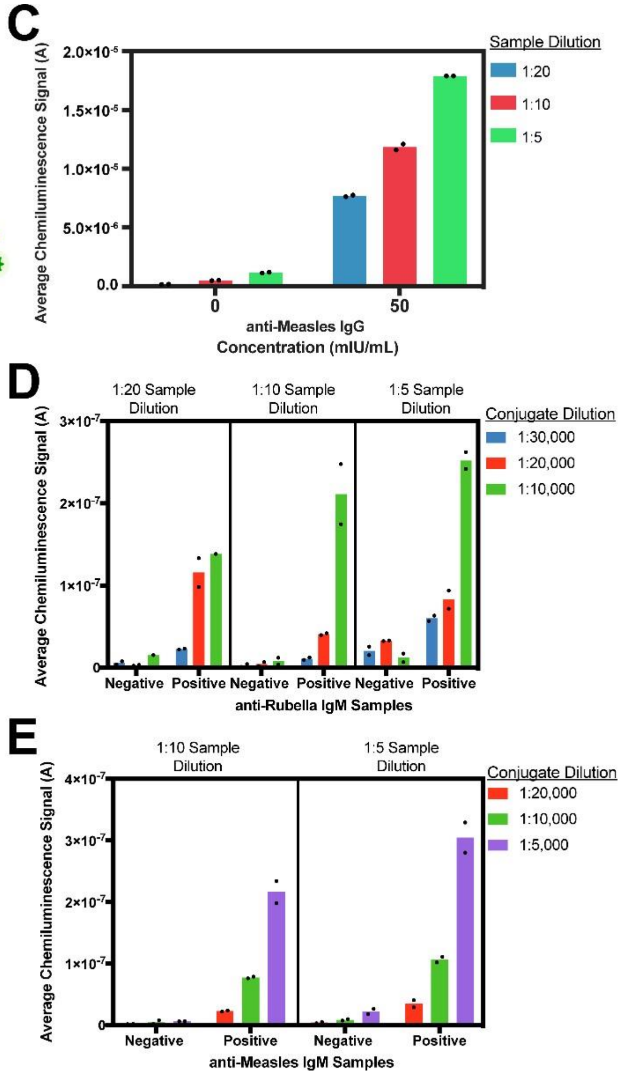

S4 Fig. MR Box v2 assay development and optimization. (A) Cartoon schematic of custom assays for MV and RV IgG (top, blue) and IgM (bottom, green). Each type of assay relies on magnetic particles bearing bound to viral antigens (brown/grey) and detection antibodies conjugated to peroxidase (yellow/red or pink/red) to catalyze the reaction of the chemiluminescent substrate luminol (green) with hydrogen peroxide. Samples in IgM assays were additionally pre-treated to remove IgG, and all assays included extensive wash-steps (not shown).

(B) A table containing all terms (factors and their interactions) from the second-order model used to fit the definitive screening DOE for the RV IgG assay. The estimates, standard error, and t-Ratio of the coefficients for these factors are included, with the terms listed in order of increasing probability. **(C)** Plot of average chemiluminescence signal in MV IgG assays with negative (left) and positive (right) spiked ADW blood samples after diluting the samples to different levels (1:20 – blue, 1:10 – red, and 1:5 – green). **(D)** Plot of average chemiluminescence signal in RV IgM assays in negative (left) and positive (right) spiked ADW blood samples after diluting the samples to different levels (1:20 – left panel, 1:10 – middle panel, 1:5 – right panel) and exposure to different dilutions of conjugate (1:30,000 – blue, 1:20,000 – red, 1:10,000 – green). **(E)** Plot of the average chemiluminescence signal in MV IgM assays in negative (left) and positive (right) spiked ADW blood samples after diluting the samples to different levels (1:10 – left panel, 1:5 – right panel) and exposure to different dilutions of conjugate (1:20,000 – red, 1:10,000 – green, 1:5,000 – purple). The coloured bars in (C-E) are averages of two replicates, with individual results shown as dots (•).

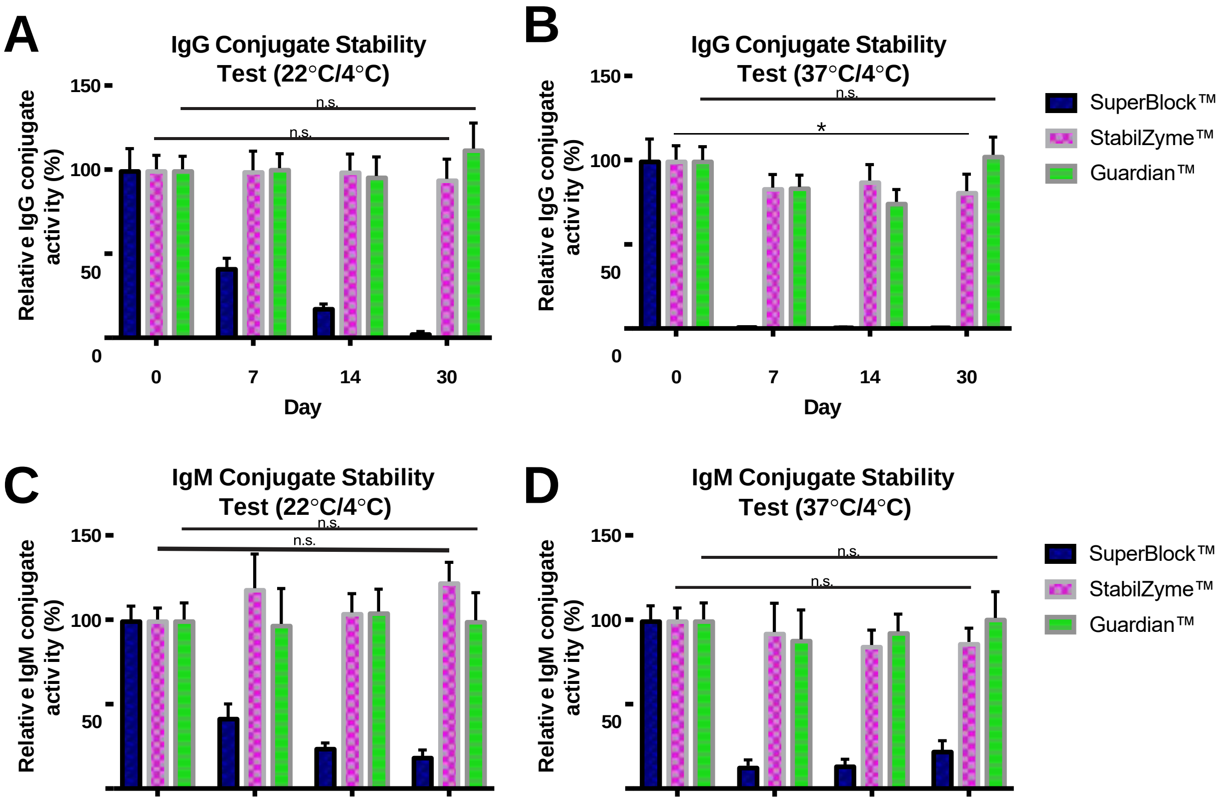

S5 Fig. Stability assessment of secondary antibody-enzyme conjugate. Plots of peroxidase activity (determined by absorbance measurements of a colorimetric substrate) normalized to the activity of conjugate stored at 4°C in SuperBlock™ for IgG **(A-B)** and IgM conjugates **(C-D)** stored in SuperBlock™ (blue), StabilZyme™ (pink), or Guardian™ (green) at 22°C or 37°C for 0, 7, 14, or 30 days. Data are averages of n=3 replicates, and error bars represent ±1 standard deviation. *$p < 0.05$, **$p < 0.01$, n.s. = $p > 0.05$.

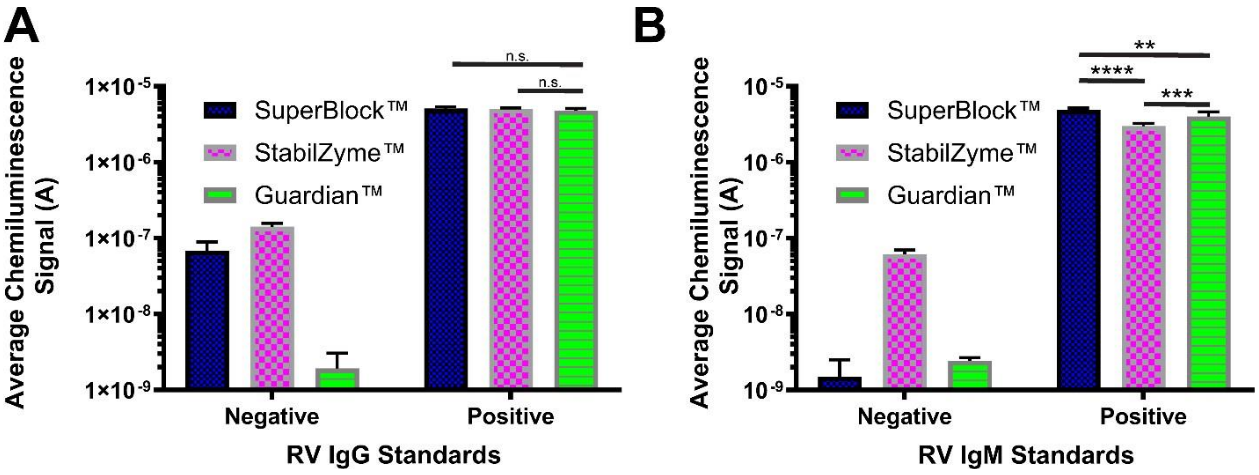

S6 Fig. Comparison of conjugate diluents on MR Box v2 assay performance. Graphs of chemiluminescent immunoassay signal measured in MR Box v2 for negative (left) and positive (right) ADW blood samples evaluated with conjugate dissolved in SuperBlock™ (blue), StabilZyme™ (pink), or Guardian™ (green) for RV **(A)** IgG and **(B)** IgM assays. Data are averages of $n \geq 2$ replicates and error bars represent ±1 standard deviation.
**$p < 0.01$, ***$p < 0.001$, ****$p < 0.0001$, n.s. = $p > 0.05$.

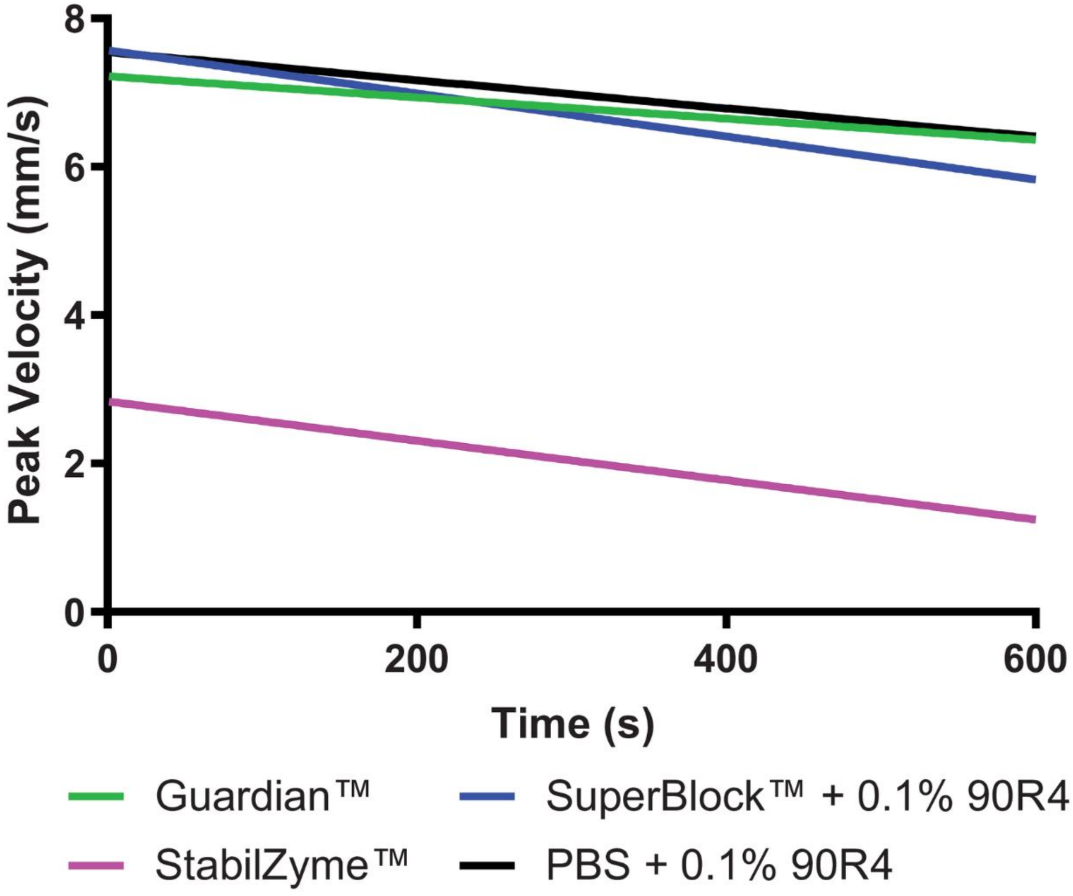

S7 Fig. Velocity data for different conjugate diluents. Plot of peak velocity versus time for measured for double-unit droplets of wash buffer (black), SuperBlock™ (blue), StabilZyme™ (pink), and Guardian™ (green) driven continuously around electrodes in DMF cartridges for 10 minutes.

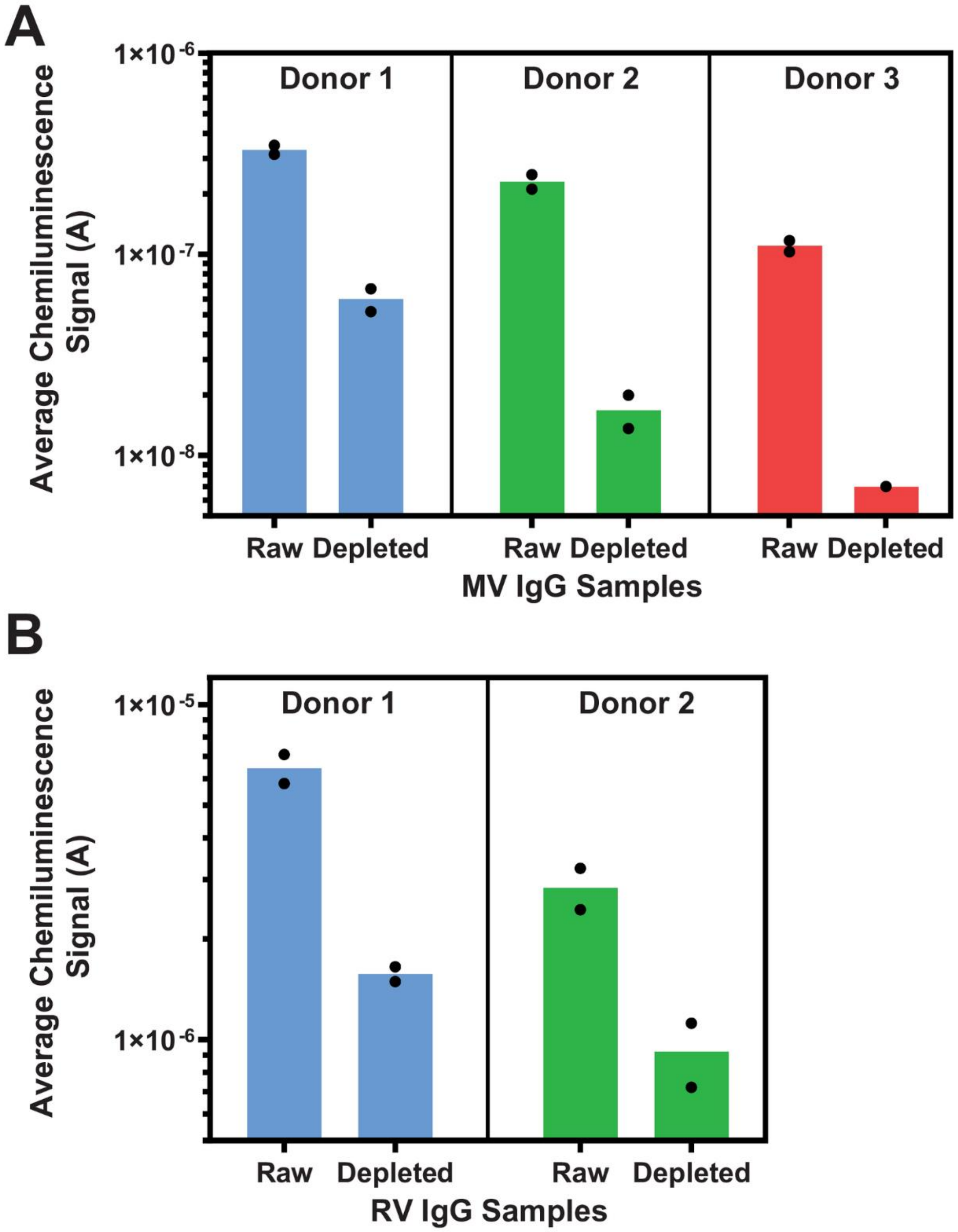

S8 Fig. Validation of optimized MR Box v2 IgG assays in the laboratory in whole blood samples. Graphs of chemiluminescent signal measured in MR Box v2 for samples evaluated by the optimized (A) MV IgG assay and the (B) RV IgG assay. Raw whole blood (left) and ADW blood (right) samples were collected from volunteers (blue, green, and red), and the bars are averages of 2 replicates, with individual results shown as dots (•)

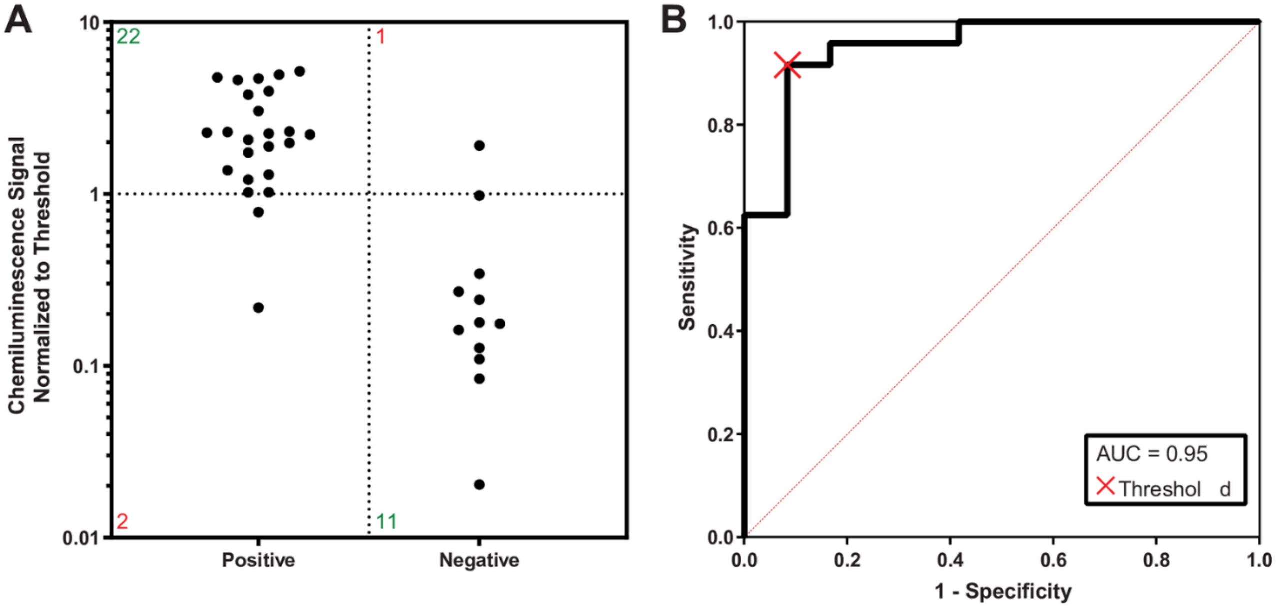

S9 Fig. Validation of optimized MR Box v2 MV IgM assay (in the laboratory) with plasma samples. **(A)** Vertical scatterplot (left) of MR Box v2 signals for samples determined to be positive or negative for MV IgM by reference tests (n = 36). Green and red numbers in the vertical scatterplots represent the number of correctly and incorrectly categorized samples by the MR Box v2, respectively. **(B)** ROC curve (right) with AUC of 0.95 and threshold (X) selected for sensitivity of 92% (95% CI, 73 to 99%), specificity of 92% (95% CI, 62 to 100%),
and overall agreement of 92% (95% CI, 78 to 98%).

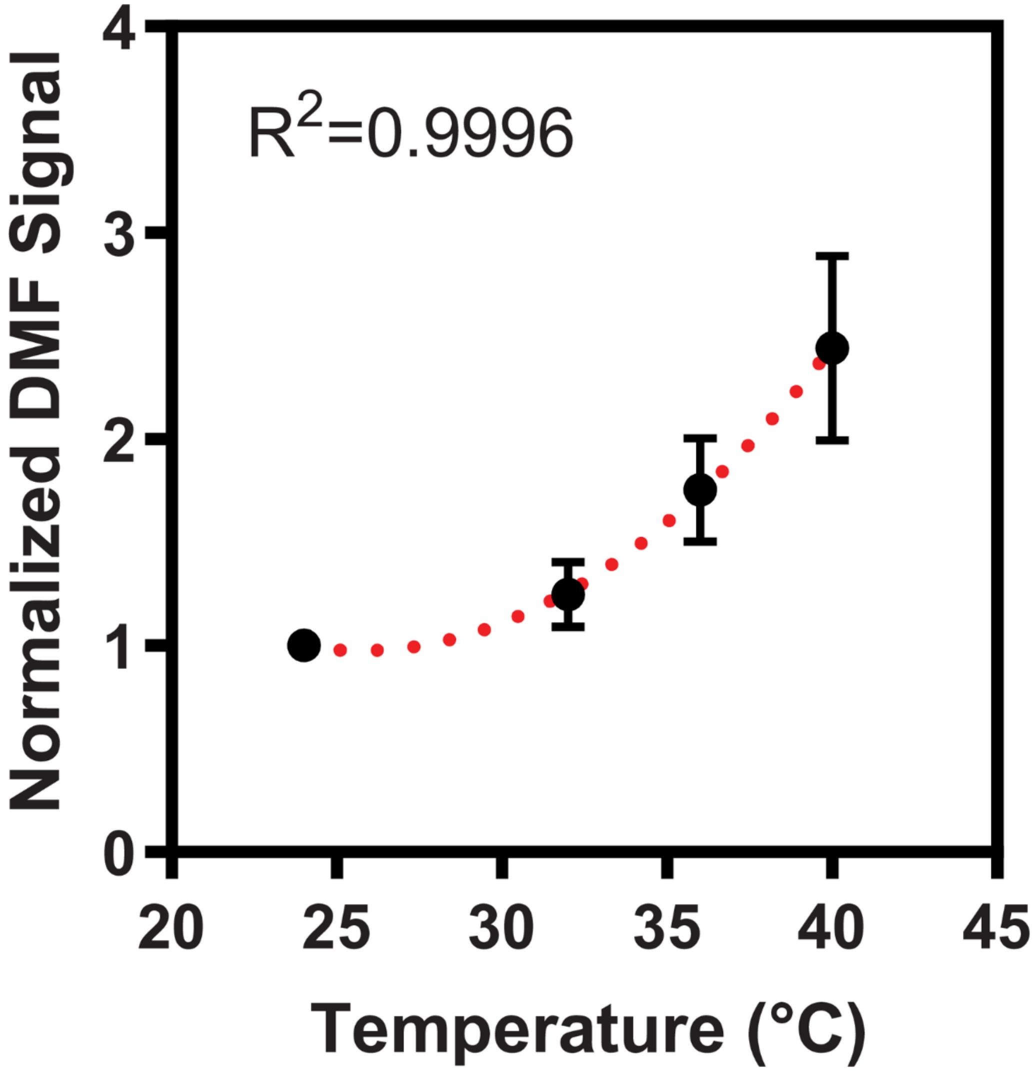

S10 Fig. Effect of temperature on MR Box v2 chemiluminescence signal. Plot of normalized peroxidase-catalyzed chemiluminescence signal as a function of temperature for the luminol/peroxide reaction used in the RV and MV IgM and IgG assays. The entire MRBox v2 instrument was enclosed in an oven, and the signal was determined at each temperature (n=3). Error bars represent ±1 standard deviation, and the plot was fitted (red dotted line) using a quadratic equation, with R^2=0.9996.

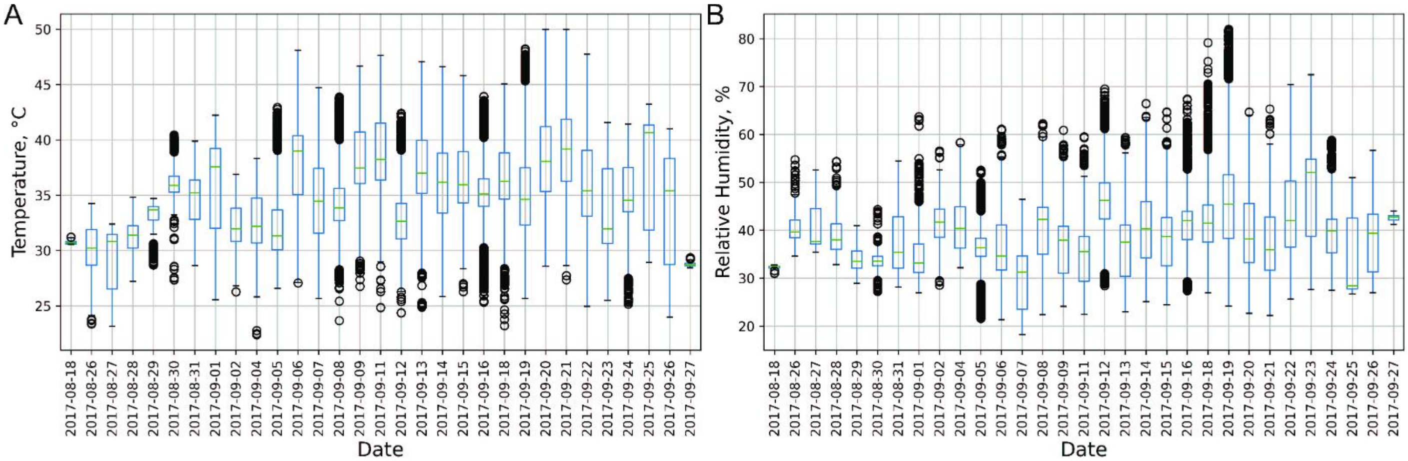

S11 Fig. Temperature and humidity data measured by the MR Box v2 instruments during the DRC field trial. (A) Box plot of temperature readings during the field trial in the DRC versus date. (B) Box plot of relative humidity readings during the field trial in the DRC versus date. Blue boxes indicate the lower and upper quantiles, center line indicates the median, and whiskers indicate the minimum and maximum values. Outliers are represented as open black circles.

Supplementary Tables

S1 Table. Bill of materials of MR-Box instrument.

Item	Quantity	Part Number	Supplier	Git Link	Price / item (USD)
DropBot Control Board v3.6	1	DB-CB-3.6-OEM	Sci-Bots	https://github.com/sci-bots/dropbot- control-board.kicad	$850
DropBot 40-channel HV Switching Board v3.1.1	3	DB-40CH-HVSB-3.1.1-OEM	Sci-Bots	https://github.com/sci-bots/dropbot- 40-channel-HV-switching-board.kicad	$850
DropBot 120-channel Connector Board v3.4	1	DB-CON-120CH-3.4- OEM	Sci-Bots	https://github.com/sci-bots/dropbot- 120-channel-pogo-pin-board.kicad	$850
Hardware Testing Board	1	120-channel test board v1.1	Sci-Bots	https://github.com/sci-bots/dropbot- v3/wiki/DropBot-Test-Board#dropbot-test-board	$40
MR Box 2 front panel board	1	N/A	Custom	https://gitlab.com/sci-bots/dropbot- front-panel.kicad	$135
Aluminum Enclosure (bottom)	1	AN-23P-02	Polycase	-	$78
Aluminum Plate	1	AN-23K	Polycase	-	$18
Plastic Lid with Metal Latch(top)	1	YH-080604-01	Polycase	-	$58
Photomultiplier tube	1	H12056-110	Hamamatsu	-	$688
PMT Mount (incl. optics)	1	N/A	Custom	-	$60
PMT Board	1	N/A	Custom	https://github.com/wheeler- microfluidics/PMT-transimpedance-amplifier	$35
RGB PMT Eval Board	1	N/A	Custom	https://microfluidics.utoronto.ca/git lab/gcc-project/RGB_LED_PMT_Test_Board	$37
Z-stage +magnetic lens	1	N/A	Custom	https://microfluidics.utoronto.ca/git lab/gcc-project/Magnet_Stage.Lab_Jack	$73
Peripheral Control Board	1	N/A	Custom	https://microfluidics.utoronto.ca/git lab/gcc-project/mr-box-peripheral-board.kicad	$51
Camera	1	ELP-USB8MP02G-L75- CA	Amazon	-	$83
LED Lights	1	24 SMD 3528 Panel	Amazon	-	$10
				Total	$5,616

S2 Table. Bill of materials of motorized Z-stage.

Item	Quantity	Part Number	Supplier	Description	Price / item (USD)
PLA	140 g	PLA+	eSUN (filaments.ca)	1 Kg<br>2.85mm Black PLA	$0.02
M2.5 Socket Head Screw	2 pcs	91292A114	McMaster-Carr	12 mm Long	$0.05
M3 Shoulder Screw	6 pcs	90265A118	McMaster-Carr	4mm Diameter, 6mm Long Shoulder	$2.48
M3 Socket Head Screw	8 pcs	91292A016	McMaster-Carr	12 mm Long	$0.05
M3 Socket Head Screw	4 pcs	91292A112	McMaster-Carr	8 mm Long	$0.04
M5 Flat Head Screw	2 pcs	92125A210	McMaster-Carr	12 mm Long	$0.12
M3 Lock Washer	6 pcs	93925A240	McMaster-Carr	Internal-Tooth	$0.03
M2.5 Hex Nut	2 pcs	91828A113	McMaster-Carr	0.45 mm Thread	$0.06
M3 Hex Nut	18 pcs	91828A211	McMaster-Carr	0.5 mm Thread	$0.06
M5 Hex Nut	2 pcs	90591A260	McMaster-Carr	0.8 mm Thread	$0.03
Push-In Blind Rivet	4 pcs	90136A552	McMaster-Carr	Nylon Split Shank, 0.115 "<br>Hole Diameter, 0.235"<br>Material Thickness	$0.06
Brass Flange Nut	1 pc	X000ZHLWFL<br>(Bar code)	Anycubic<br>(amazon.com)	Diameter 8mm; Pitch 2mm	$2.50
T8 Lead Screw	1 pc	3D printer accessories kit	GOCHANGE<br>(amazon.ca)	500 mm Long<br>Pitch 2mm<br>(Cut in 100 mm)	$4.39
4mm To 8mm Shaft Coupler	1 pc	N/A	amazon.ca	19 x2 5 mm	$2.54
Ball Bearing Pillow Block	1 pc	9978	Flyouth (amazon.ca)	Bore diameter: 8mm (KP08)	$4.87
Stepper Motor	1 pc	EM-483	Recycled Epson Printers	Max Current 1.4A, Step 1.8°	$0.00
				Total	$34.56

S3 Table. Summary of conditions tested in DOE analysis for optimization of four whole blood immunoassays on DMF.

Conditions Tested	RV IgG	MV IgG	RV IgM	MV IgM
Pre-Processing – sample dilution level	1:20, 1:10, 1:5	1:20, 1:10, 1:5	1:20, 1:10, 1:5	1:10, 1:5
Assay Step 2 – Sample incubation time with particles	3, 5, 7 minutes	N/A	N/A	N/A
Assay Steps 4-9 –Number of post sample-incubation wash steps	2, 4, 6 washes	2, 4, 6 washes	N/A	N/A
Assay Step 10 – Conjugate dilution level (relative to stock solutions) prior to loading onto cartridge	N/A	N/A	1:30k, 1:20k, 1:10k	1:20k, 1:10k, 1:5k

S4 Table. Multiplicative (f_m) and subtractive (f_s) factor values for each condition used to normalize the field trial results.

Factor	Group	f_m	f_s
Box ID	MR Box v2 1	5.43267593	3.89241E-07
	MR Box v2 2	0.009590374	-2.63841E-07
	MR Box v2 3	1.218086149	3.03216E-08
	MR Box v2 4	0.218640408	-1.55721E-07
Humidity	(0.3, 0.35]	16.00874113	1.02136E-06
	(0.35, 0.4]	0.793406493	-3.71865E-08
	(0.4, 0.45]	0.501772696	-9.9254E-08
	(0.45, 0.5]	1.578417941	8.72438E-08
	(0.5, 0.55]	0.264371052	-1.65342E-07
	(0.55, 0.6]	0.056017495	-2.5978E-07
	(0.7, 0.75]	0.000626759	-3.31809E-07
	Nan	0.135113627	-2.15232E-07
Temperature	(26, 28]	0.000392561	-4.21507E-07
	(28, 30]	2.062910921	1.87614E-07
	(30, 32]	0.821167771	-4.03439E-08
	(32, 34]	5.373146304	5.66779E-07
	(34, 36]	0.046356258	-3.37441E-07
	(36, 38]	0.134477013	-2.72332E-07
	(38, 40]	2.929574616	3.06008E-07
	(40, 42]	3.01089512	3.16153E-07
	Nan	0.084626306	-3.0493E-07
Cartridge Type	photolith./std.	0.532375	4.49741E-07
	photolith./PET-ITO	1.187241	-1.8008E-07
	printed/std.	1.280385	-2.6966E-07

S5 Table. Summary of key improvements of MRBox v2 with respect to its predecessor MRBox v1.

Key Features	MRBox v1	MRBox v2	Comments
**Enclosure**	Acrylic	Aluminum Base & Polycarbonate Lid	Upgraded enclosure is more robust, suitable for frequent travel.
**Photomultiplier Tube (PMT)**	Standard	Gate Function	Gate function acts as an electronic shutter, protecting the PMT from excessive light when the lid is open, while eliminating the need for long PMT warm up periods.
**PMT Mounting**	Lid (Moving)	Pogo-pin Board (Stationary)	In the new version of the MRBox the PMT is fixed on the upper side of the DMF cartridge allowing for more precise alignment of the PMT with the chemiluminescence reading zone.
**Current Sensor**	Dstat	Dedicated PMT Board	The dedicated current sensing board mounts directly onto the PMT and converts the signal from analog to digital allowing for lossless data transfer and easier operation.
**Magnet Actuator**	Servo	Stepper powered Z-stage	Z-stage is only powered when switching between states, and thus greatly reduces the overall power consumption.
**Power**	110/220V	12V Battery	Upgraded electronics allow the instrument to run on a 12V battery for more than 8 hours.
**Hardware Testing**	N/A	Capacitor Board	The capacitor board allows for periodical testing of the DMF hardware, to ensure performance between days, as well as provide guidance during troubleshooting.
**Camera**	HD Pro C920	USB8MP02G-L75-CA	Lower cost camera and improved image quality
**Cartridge alignment**	Manual	Automatic	New Push to Load (P2L) mechanism allows for automatic chip alignment and loading to the DMF hardware, making it trivial to load and unload the DMF cartridges.

Supplementary References

1. Ng AH, Fobel R, Fobel C, et al. A digital microfluidic system for serological immunoassays in remote settings. Science translational medicine **2018**; 10:eaar6076.

2. Rackus DG, Dryden MD, Lamanna J, et al. A digital microfluidic device with integrated nanostructured microelectrodes for electrochemical immunoassays. Lab on a Chip **2015**; 15:3776-84.

3. Dixon C, Ng AH, Fobel R, Miltenburg MB, Wheeler AR. An inkjet printed, roll-coated digital microfluidic device for inexpensive, miniaturized diagnostic assays. Lab on a Chip **2016**; 16:4560-8.

4. Ng AH, Lee M, Choi K, Fischer AT, Robinson JM, Wheeler AR. Digital microfluidic platform for the detection of rubella infection and immunity: a proof of concept. Clinical chemistry **2015**; 61:420-9.

5. Swyer I, Fobel R, Wheeler AR. Velocity saturation in digital microfluidics. Langmuir **2019**; 35:5342-52.

6. Virtanen P, Gommers R, Oliphant TE, et al. SciPy 1.0: fundamental algorithms for scientific computing in Python. Nature methods **2020**; 17:261-72.

7. Harris CR, Millman KJ, Van Der Walt SJ, et al. Array programming with NumPy. Nature **2020**; 585:357-62.

8. McKinney W. Data structures for statistical computing in python. In: Proceedings of the 9th Python in Science Conference. Austin, TX:51-6.

9. Pedregosa F, Varoquaux G, Gramfort A, et al. Scikit-learn: Machine learning in Python. the Journal of machine Learning research **2011**; 12:2825-30.

10. Hamamatsu PK. PHOTOMULTIPLIER TUBES. Basics and Applications THIRD EDITION (Edition 3a. **2007**.

11. Jones B, Nachtsheim CJ. A class of three-level designs for definitive screening in the presence of second-order effects. Journal of Quality Technology **2011**; 43:1-15.

12. Roda A. Chemiluminescence and bioluminescence: past, present and future. Royal Society of Chemistry, **2011**.

13. Sklavounos AA, Lamanna, J., Modi, D., Gupta, S., Mariakakis, A., Callum, J., & Wheeler, A.R. Digital Microfluidic Hemagglutination Assays for Blood Typing, Donor Compatibility Testing, and Hematocrit Analysis. **2021**.

14. Boess F, Boelsterli UA. Luminol as a probe to assess reactive oxygen species production from redox-cycling drugs in cultured hepatocytes. Toxicology mechanisms and methods **2002**; 12:79-94.

15. Michael S, Auld D, Klumpp C, et al. A robotic platform for quantitative high-throughput screening. Assay and drug development technologies **2008**; 6:637-57.

16. Nielsen UB, Geierstanger BH. Multiplexed sandwich assays in microarray format. Journal of immunological methods **2004**; 290:107-20.
